# Impaired Mitochondrial Energy Production Causes Light-Induced Photoreceptor Degeneration Independent of Oxidative Stress

**DOI:** 10.1371/journal.pbio.1002197

**Published:** 2015-07-15

**Authors:** Manish Jaiswal, Nele A. Haelterman, Hector Sandoval, Bo Xiong, Taraka Donti, Auinash Kalsotra, Shinya Yamamoto, Thomas A. Cooper, Brett H. Graham, Hugo J. Bellen

**Affiliations:** 1 Department of Molecular and Human Genetics, Baylor College of Medicine (BCM), Houston, Texas, United States of America; 2 Howard Hughes Medical Institute, BCM, Houston, Texas, United States of America; 3 Program in Developmental Biology, BCM, Houston, Texas, United States of America; 4 Department of Pathology and Immunology, BCM, Houston, Texas, United States of America; 5 Jan and Dan Duncan Neurological Research Institute, Texas Children’s Hospital (TCH), Houston, Texas, United States of America; 6 Department of Neuroscience, BCM, Houston, Texas, United States of America; New York University, United States of America

## Abstract

Two insults often underlie a variety of eye diseases including glaucoma, optic atrophy, and retinal degeneration—defects in mitochondrial function and aberrant Rhodopsin trafficking. Although mitochondrial defects are often associated with oxidative stress, they have not been linked to Rhodopsin trafficking. In an unbiased forward genetic screen designed to isolate mutations that cause photoreceptor degeneration, we identified mutations in a nuclear-encoded mitochondrial gene, *ppr*, a homolog of human *LRPPRC*. We found that *ppr* is required for protection against light-induced degeneration. Its function is essential to maintain membrane depolarization of the photoreceptors upon repetitive light exposure, and an impaired phototransduction cascade in *ppr* mutants results in excessive Rhodopsin1 endocytosis. Moreover, loss of *ppr* results in a reduction in mitochondrial RNAs, reduced electron transport chain activity, and reduced ATP levels. Oxidative stress, however, is not induced. We propose that the reduced ATP level in *ppr* mutants underlies the phototransduction defect, leading to increased Rhodopsin1 endocytosis during light exposure, causing photoreceptor degeneration independent of oxidative stress. This hypothesis is bolstered by characterization of two other genes isolated in the screen, *pyruvate dehydrogenase* and *citrate synthase*. Their loss also causes a light-induced degeneration, excessive Rhodopsin1 endocytosis and reduced ATP without concurrent oxidative stress, unlike many other mutations in mitochondrial genes that are associated with elevated oxidative stress and light-independent photoreceptor demise.

## Abstract 2

Some mitochondrial disorders cause blindness through increased oxidative stress. This study shows that in other such disorders, light-activated photoreceptors degenerate because the shortfall in mitochondrial energy production impairs rhodopsin trafficking and induces toxicity.

## Author Summary

Mitochondrial dysfunction is associated with a number of metabolic and neurological diseases such as Leigh syndrome and progressive blindness. Increased oxidative stress, which is often associated with mitochondrial dysfunction, is thought to be a common cause of disease progression. Here, we identified nuclear genes that encode mitochondrial proteins, whose loss causes the demise of photoreceptor neurons. Contrary to the common idea that this degeneration is triggered by elevated levels of oxidative stress, we find no change in the levels of oxidative stress. We show that activating photoreceptor neurons with light significantly increases energy production, and that this process is required to sustain their activity. Mitochondrial dysfunction impairs this capacity and leads to a premature termination of the light response. This in turn impairs the cycling of the light-sensitive receptor Rhodopsin in photoreceptors, and Rhodopsin accumulates in the cell inducing toxicity. This distinct mechanism of degeneration suggests that different mitochondrial diseases may follow different paths of disease progression and would hence respond differently to treatments.

## Introduction

The causes of progressive dysfunction or death of photoreceptors (PRs) is genetically heterogeneous in humans [1]. PR degeneration is a complex process influenced by numerous genes and environmental factors. Although prolonged exposure to sunlight is one of the major causes of retinal degeneration, more than 200 genes have been associated with retinal diseases in humans [2,3]. Genes associated with retinal diseases affect a variety of cellular processes including phototransduction, cellular stress, metabolism, catabolism, and mitochondrial function [1,3,4]. PR activity is a highly energy-dependent process [5,6], and mitochondrial dysfunction has been implicated in glaucoma, optic atrophy, Leber hereditary optic neuropathy (LHON), and retinitis pigmentosa [1,7,8]. A widely accepted view postulates that increased reactive oxygen species (ROS) levels, resulting from mitochondrial dysfunction, is a major cause of retinal degeneration in human and mouse [9]. According to this model, light triggers mitochondrial activity, leading to increased production of ROS and cellular damage.

In *Drosophila*, the function of several mitochondrial genes has been assessed in PRs. These include Succinate dehydrogenase A (SdhA), a subunit of mitochondrial Complex II [10], Sicily, a protein required for Complex I assembly in mitochondria [11], Opa1, a protein required for inner mitochondrial membrane fusion [12], Aats-met, a mitochondrial methionyl-tRNA synthetase [13], and NnaD, a mitochondrial zinc carboxypeptidase [14]. Consistent with previously published data in mammals [9], all of the mutants in which ROS was assessed have been associated with elevated ROS, suggesting that increased oxidative stress promotes PR degeneration [10–13].

Genetic screens in *Drosophila* have identified mutations in numerous genes that cause PR degeneration and that are also conserved in human. These mutants can be categorized into two broad groups: those that cause light- and activity-dependent PR degeneration and those that cause light- and activity-independent degeneration. The majority of mutations in genes that are primarily implicated in the phototransduction pathway typically cause light-dependent PR degeneration either due to aberrant Rhodopsin1 (Rh1) trafficking or Ca^2+^-mediated excitotoxicity [15–17]. However, mutations that constitutively activate the phototransduction pathway, leading to excessive Ca^2+^ influx, cause light-independent PR degeneration, e.g., loss of function of *rdgA* [18] or in *trp* ^*P365*^, which encodes a constitutively active TRP (Transient Receptor Potential) channel [19].

Light-independent PR degeneration has also been documented for a single fly mitochondrial mutant. The authors showed that the demise of neurons is due to oxidative stress because of the loss of *SdhA* in mitochondria [10]. Since light dependence has not been tested for the other mutations causing mitochondrial dysfunctions, it is not obvious which mutations cause which type of neurodegeneration, nor what the nature of the insults are that underlie these neurodegenerations.

In this study, we show that mutations that impair mitochondrial ATP production without a concurrent increase in oxidative stress exhibit light-dependent PR degeneration. In contrast, mutations that affect ATP production as well as oxidative stress exhibit light-independent PR degeneration that can be exacerbated by light exposure. Furthermore, the observed light-induced PR degeneration in mutants affecting mitochondrial ATP synthesis stems from defects in the phototransduction cascade leading to aberrant endocytosis and delay in the degradation of Rh1.

## Results

### Ppr Localizes to Mitochondria and Its Loss Causes a Progressive Defect in ERGs

**Fig 1:**
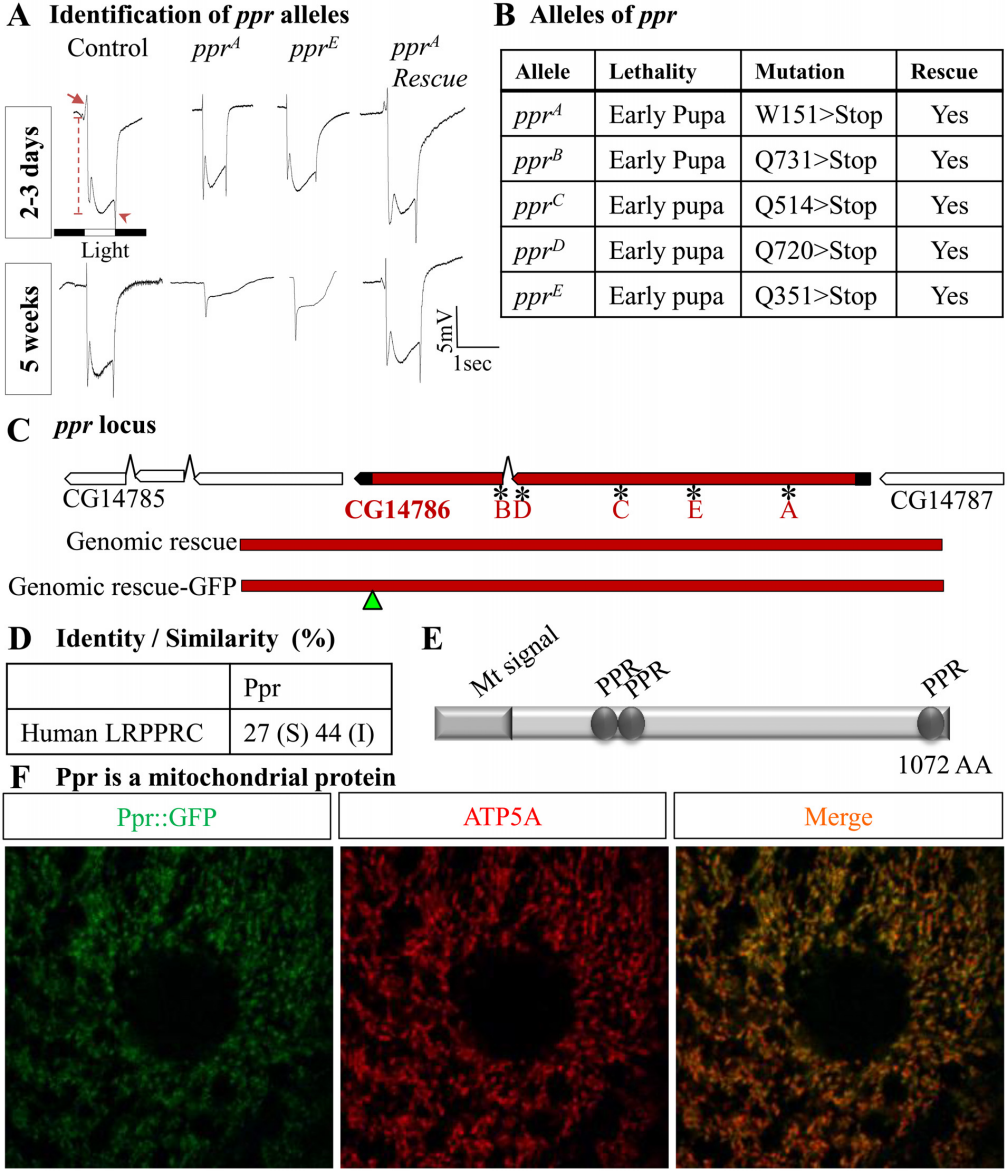
Mutations in *ppr* cause PR degeneration. (A) ERG traces from eye clones of control (*y w* FRT 19A), *ppr* ^*A*^, *ppr* ^*E*^, and *ppr* ^*A*^-carrying genomic transgene (CH322-75O21)-containing wild-type *ppr* (*ppr* ^*A*^ *Rescue*). Arrow and arrowhead indicate “on” and “off” transient respectively. Dashed line indicates the amplitude. Top: upon eclosion, flies were raised in dark for 2–3 d, after which ERGs were recorded. Bottom: flies were reared for 5 wk in a 12 h light/12 h dark cycle. (B) Table presenting all identified *ppr* alleles, their lethal stage, and molecular lesion. Lethality in these alleles is rescued by a genomic copy of *ppr* or ubiquitous expression of *ppr* cDNA. (C) Genomic location of the *ppr* (*CG14786*) gene, displaying the position of the molecular lesions associated with the different alleles. The genomic rescue construct spans a region from 0.3 kb upstream to 1.3 kb downstream of the *ppr* coding region. The genomic rescue-Green Fluorescent Protein (GFP) construct contains a GFP tag at the C-terminus of *ppr*. (D) The Ppr protein has 27% identity (I) and 44% similarity (S) to human LRPPRC. (E) The Ppr protein’s predicted mitochondrial localization signal and PPR repeats are shown. (F) Colocalization of the GFP-tagged Ppr protein (green) with mitochondrial complex V (ATP5A antibody, red) in larval muscle.

To identify genes required for the maintenance of neurons in the visual system, we performed an unbiased mosaic genetic screen on the X chromosome. We induced large homozygous mutant clones of essential genes in the eyes using the *ey*-FLP system and screened for age-dependent defects in electroretinograms (ERGs) [20,21]. ERG recordings are induced by light and exhibit “on” and “off” transients (arrow and arrowhead in Fig 1A), indicative of synaptic communication between the PR neurons and postsynaptic cells. They also exhibit a corneal negative response, the amplitude of which signifies the depolarization of PR neurons (dashed line) (Fig 1A). One of the isolated complementation groups, named *ppr*, *p* *entatrico* *p* *eptide* *r* *epeat containing protein* (see below), displayed a dramatic reduction in ERG amplitude as well as a loss of “on” and “off” transients in five-wk-old but not 2–3-d-old animals, suggesting a progressive PR degeneration (Fig 1A).

The causative mutations of the five alleles of this complementation group were mapped to *CG14786 (ppr)*, an uncharacterized gene in *Drosophila* (Fig 1B and 1C and S1 Fig). All alleles carry a premature stop codon (Fig 1B and 1C). Two rescue transgenes, a 20 kb P[acman] BAC (P/ΦC31 artificial chromosome for manipulation) CH322-75O21 genomic fragment that contains *CG14786* [22] and a 5 kb genomic fragment of *CG14786* (Fig 1C), rescue the pupal lethality associated with the loss of *ppr*. Moreover, *ppr* mutants (*ppr* ^*A*^, W150Stop) carrying the genomic rescue transgene (P[acman] BAC CH322-75O21, S1A Fig) show normal ERG amplitudes in aged animals (Fig 1A).

The human homolog of *ppr* is *LRPPRC*, a mitochondrial protein (Fig 1D) whose loss causes Leigh syndrome [23]. Similar to LRPPRC and other pentatricopeptide proteins, the Ppr protein contains multiple PPR repeats (Fig 1E and S1B Fig; hence *ppr*). The Ppr protein has a putative amino terminal mitochondrial targeting sequence, as predicted by Mitoprot [24] (Fig 1E). To assess the subcellular localization of the protein, we created transgenic lines carrying a 5 kb genomic rescue transgene in which *ppr* is tagged at the C-terminus with Green Fluorescent Protein (GFP) (Fig 1C). This construct rescues the lethality of *ppr* ^*A*^ and *ppr* ^*E*^, is ubiquitously expressed, and the protein colocalizes with a mitochondrial protein, ATP5A (Fig 1F and S1C and S1D Fig). In summary, we identified mutations in a fly homolog of LRPPRC, a protein that is localized to mitochondria and whose loss causes a progressive decline of PR function.

### Loss of *ppr* Results in Light-Dependent PR Degeneration

**Fig 2:**
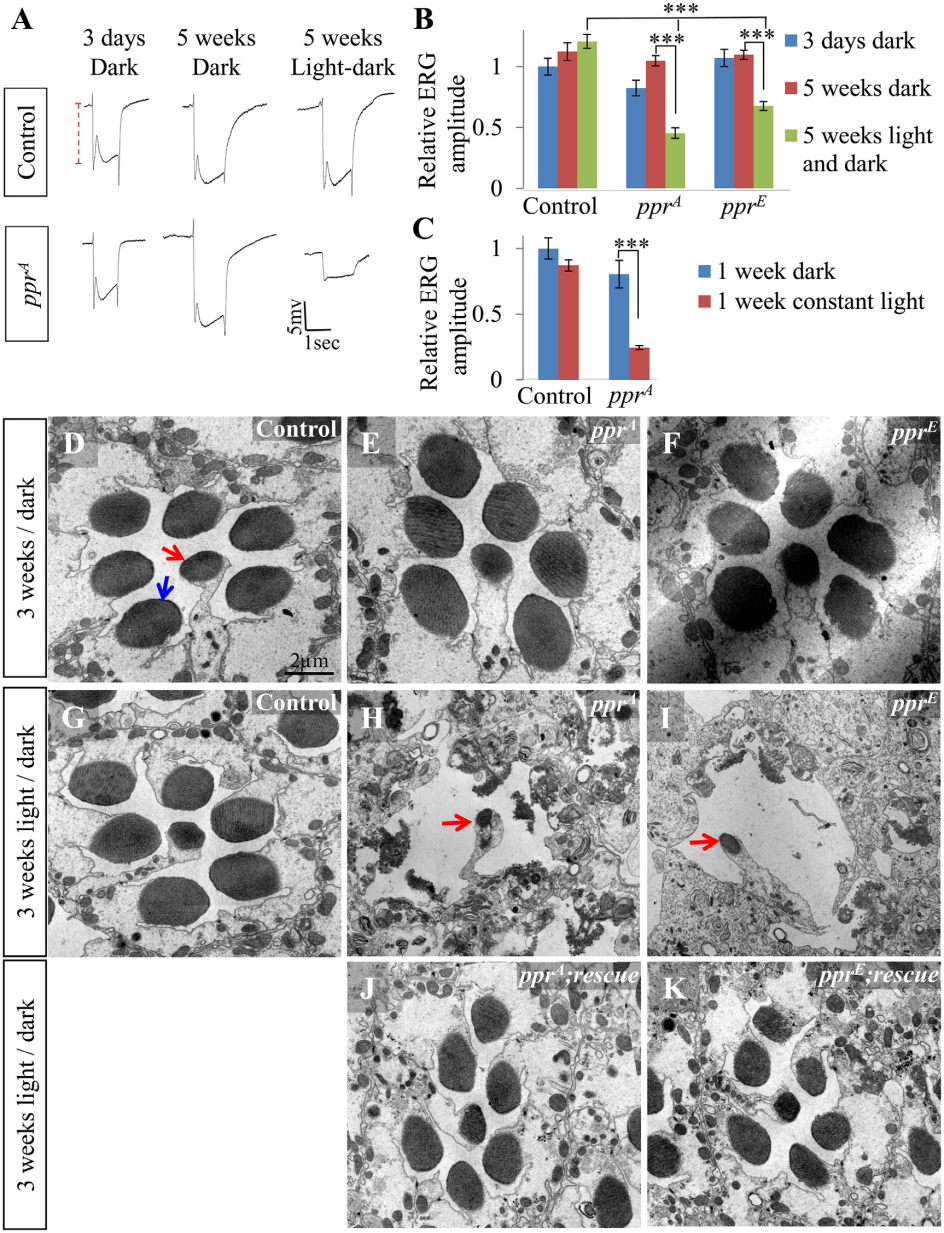
PR degeneration due to *ppr* loss of function is light-dependent. (A) ERG traces from control and *ppr* ^*A*^ mutant eye clones. Flies were raised either in constant dark for three days, constant dark for five weeks, or a 12 h light/dark cycle for five weeks. Dashed line indicates ERG amplitude. (B) Quantification of ERG amplitudes for experiment shown in (A). (C) Quantification of the ERG amplitude of control and *ppr* ^*A*^ mutant eye clones of flies that were grown in dark or constant light for one week. Error bars represent mean ± standard error of the mean (SEM); statistical significance was determined using a two-tailed Student’s *t* test (*p*-value: *** < 0.001). (D–K) Transmission Electron Microscopy (TEM) of a single ommatidium from control (D, G), *ppr* ^*A*^ (E, H), *ppr* ^*E*^ (F, I), *ppr* ^*A*^; genomic rescue (J) and *ppr* ^*E*^; genomic rescue (K). Flies were raised in the dark (D–F) or in a 12 h light/dark cycle for three weeks (G–K). The dark structures indicated by arrows in (D) are rhabdomeres. The red arrow specifically indicates R7 or R8 rhabdomeres, which do not degenerate in mutants exposed to light (H, I). Light intensity during light periods was ~1,800 Lux.

To determine whether the progressive age-dependent decay in ERG amplitudes is light-dependent, we raised the flies in constant darkness or a 12 h light/dark cycle for five weeks. The ERG amplitudes of mutant PRs are not affected when the flies are raised in the dark, whereas flies maintained under a 12 h light/dark cycle exhibit severely diminished ERG amplitudes (Fig 2A and 2B). Moreover, the ERG amplitude is dramatically reduced in one-week-old *ppr* mutant flies if they are maintained under constant light (Fig 2C). Hence, the progressive defect in ERG loss in *ppr* mutants is induced by light.

To assess the morphological features of *ppr* mutant PRs upon aging and light exposure, we examined cross-sections of the retina by light and Transmission Electron Microscopy (TEM). In the fly eye, PR cells are organized in ~800 ommatidia, and each ommatidium contains eight PR cells (R1–R8). Cross-sections across the retinal PRs reveal the dense microvillar structures of the rhabdomere (Fig 2D arrows), a stack of membranes that are highly enriched in Rh1 and are required for phototransduction [15]. Retina of control, young *ppr* mutants and *ppr* mutants reared in the dark for three weeks show very similar morphological features (Fig 2D–2F and S2A–S2F Fig). Moreover, the morphology of PRs of control flies maintained on a 12 h light/dark cycle for three weeks are comparable to young flies (Fig 2G and S2G Fig), whereas the morphology of *ppr* mutants is highly aberrant (Fig 2H and 2I and S2H and S2I Fig). This phenotype is fully rescued by a genomic rescue transgene (Fig 2J and 2K). Degeneration occurs in PRs R1–R6, which all express Rh1 [25] (blue arrow in Fig 2D), whereas R7 and R8 are spared (red arrows, Fig 2H and 2I; compare to red arrow in 2D). Hence, the residual ERG amplitude in *ppr* ERG traces may be contributed by R7 and R8. In summary, a light-induced mechanism causes degeneration of PRs in *ppr* mutants.

### Loss of *ppr* Leads to a Rapidly Diminishing Light Response upon Repetitive Stimulation

**Fig 3:**
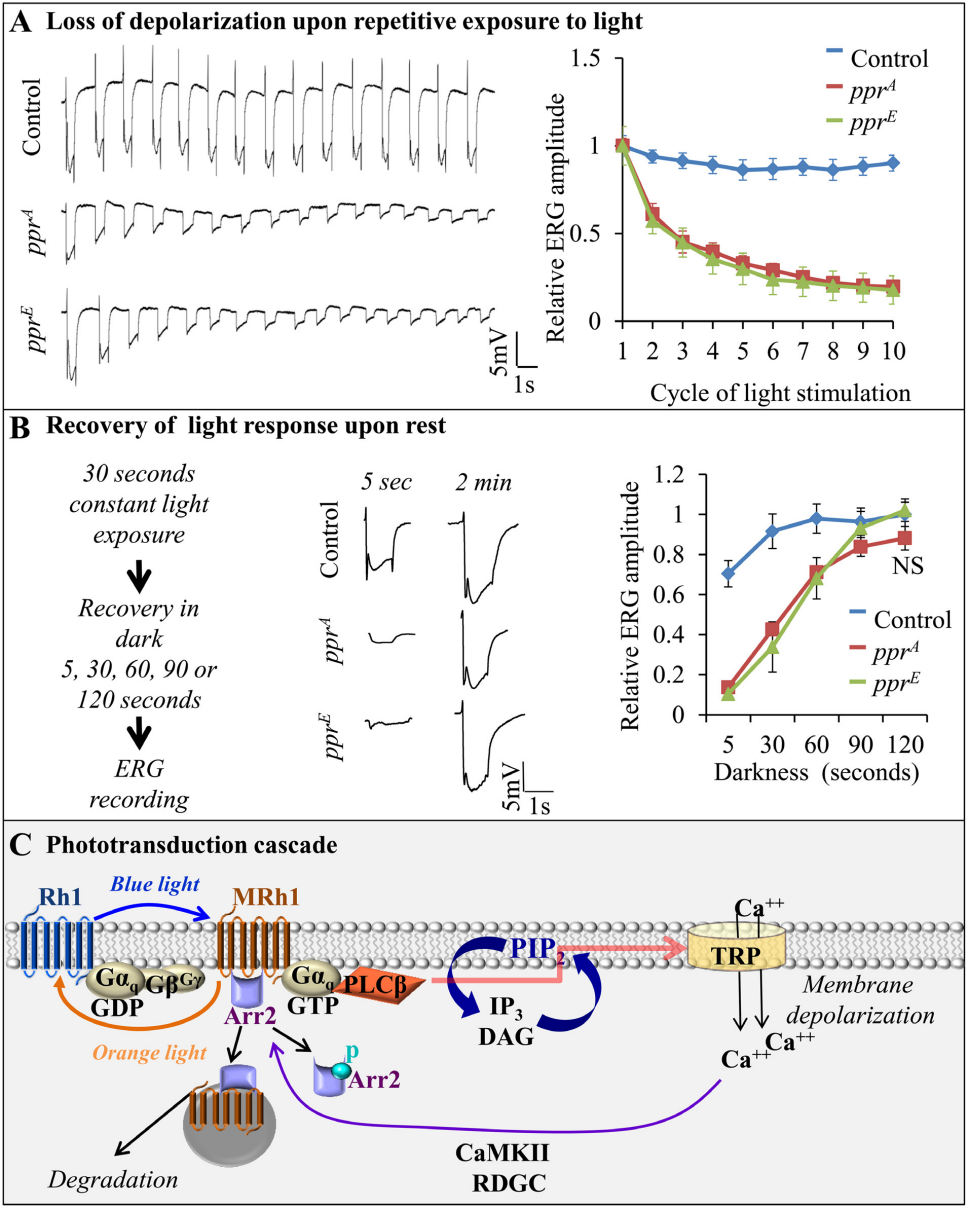
*ppr* is required to maintain PR depolarization upon repetitive stimulation. (A) ERG traces during repetitive light stimuli (1 sec light and 1.5 sec dark) recorded from eye clones of control, *ppr* ^*A*^ and *ppr* ^*E*^ (left). Quantification of the change in ERG amplitude, relative to the amplitude of the response to the initial light stimulus, is shown on the right. Error bars represent ± SEM. (B) Recovery time of ERG amplitude (light response) in control, *ppr* ^*A*^ *and ppr* ^*E*^ eye clones. Following 30 sec of light exposure (~1,700 Lux), ERG amplitudes were measured after 5, 30, 60, 90 or 120 sec of recovery in dark. ERG traces after 5 sec and 2 min are shown (middle), and quantification of the relative ERG amplitude is shown on the right. Error bars represent ± SEM; NS (two-tailed Student’s *t* test not significant). (C) Schematic presentation of the phototransduction cascade and the mechanism of Rh1 recycling and endocytosis. When exposed to blue light, Rh1 is converted to meta-Rh1 (MRh1). Through a G-protein cascade, MRh1 activates the TRP and TRPL channels, leading to a Ca^2+^ influx and PR depolarization. MRh1 is quickly phosphorylated by GPRK1 and bound by Arrestin2 (Arr2), leading to the inactivation of MRh1 [30,31]. Subsequently, MRh1 is converted to Rh1 by orange light. Rh1 is recycled through a Ca^2+^-dependent pathway leading to Arr2 released from Rh1 [32–34]. A fraction of Rh1 forms a stable complex with Arr2 when exposed to light. This complex is endocytosed and degraded by the endolysosomal system (Reviewed in [15,17]).

Although both young and old *ppr* mutants raised in the dark display normal ERGs, light-dependent PR degeneration typically indicates a defect in the phototransduction cascade [15,16]. To establish if the *ppr* mutants display defects in the phototransduction cascade, we recorded ERGs upon repetitive pulses of light [26–29]. Flies were kept in the dark for 3–4 min prior to the ERG recordings and stimulated with 10–15 cycles consisting of white light for 1 sec followed by a 1.5 sec dark period (Fig 3A). In *ppr* mutant eyes, there is a rapid run-down of the amplitude and “on” and “off” responses, whereas control flies show only a very modest reduction in amplitude (Fig 3A). Since this phenotype is activity-dependent, it prompted us to assess the inactivity period (darkness) needed to recover normal ERG amplitude in *ppr* mutant eyes. We exposed flies to light for 30 sec to maximally reduce the stimulation response in *ppr* mutant eyes (Fig 3B). Upon a 5–120 sec rest period, we measured the recovery of the ERG amplitude and observed a full recovery to the light response upon a two minute rest period in the dark (Fig 3B). These results demonstrate that Ppr function is required to maintain PR activity.

Phototransduction in *Drosophila* PRs (Fig 3C) is initiated with the conversion of Rh1 to active meta-Rh1 (MRh1) by blue light (~480 nm) [15,29,35,36]. MRh1 triggers a G-protein cascade that activates Phospholipase C (PLC, encoded by *norpA*), causing hydrolysis of the membrane phospholipid, phosphatidylinositol 4,5-bisphosphate [PI(4,5)P_2_] (Fig 3C). Hydrolysis of PI(4,5)P_2_ activates the light-sensitive TRP channel causing a Ca^2+^ influx, which is essential to depolarize the PRs [29,37–41].

**Fig 4:**
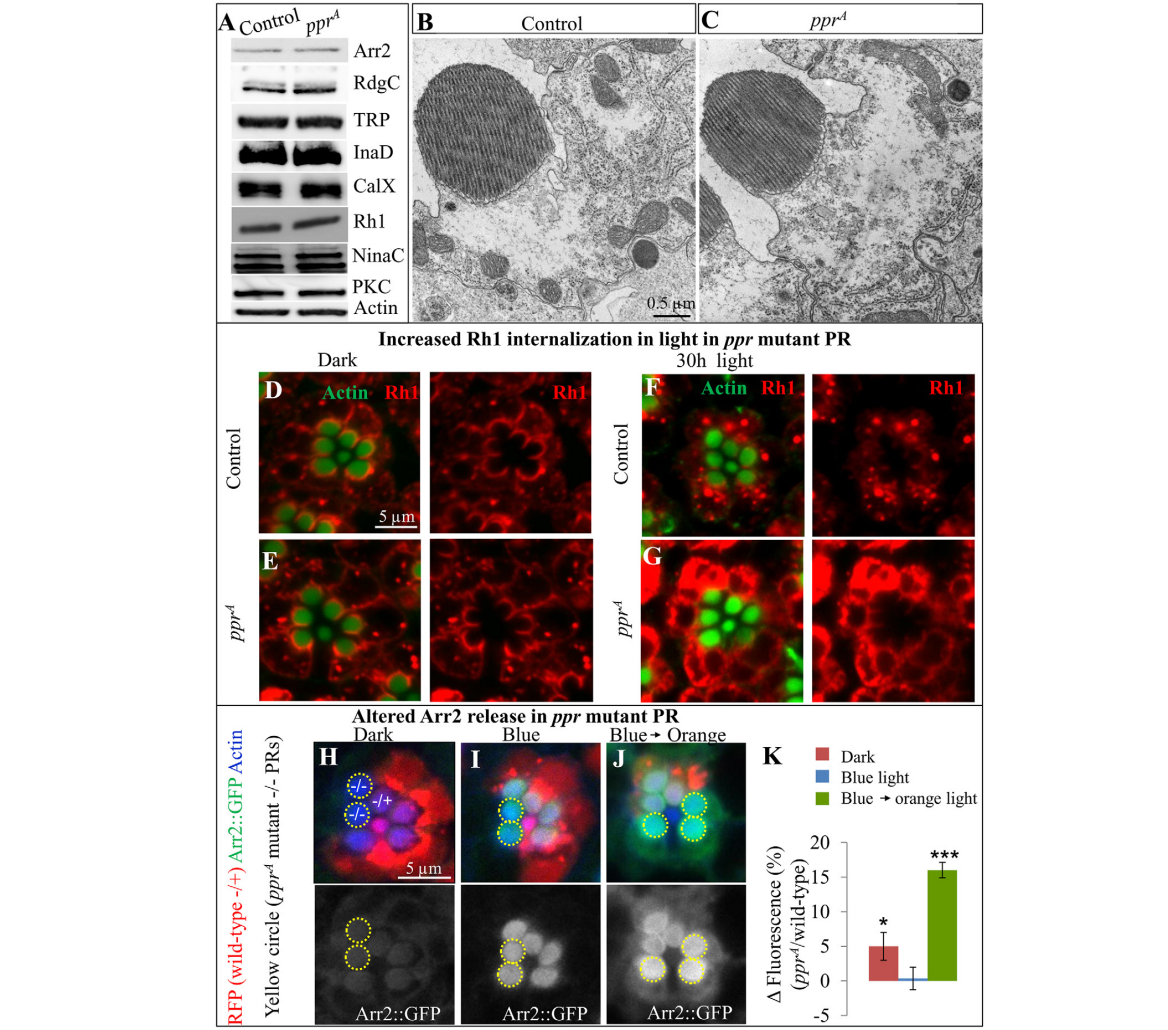
Light induces Rh1 accumulation in ppr mutant PRs. (A) Western blots of proteins regulating phototransduction. Protein was extracted from heads of flies containing control or *ppr* ^*A*^ mutant eyes. (B–C) TEM of a single PR from 2–3-d-old, dark-reared control or *ppr* ^*A*^ eye clones. (D–G) Whole mount Rh1 (red) immunostaining in control (D, F) and *ppr* ^*A*^ mutant PR (E, G). Rhabdomeres are marked by Phalloidin/Actin (green). Flies used in this experiment were 3–4 d old and raised in the dark (D, E) or exposed to ~30 h of light (F, G). (H–K) Arr2::GFP (green or grey) levels in rhabdomeres of *ppr* ^*A*^ mosaic retina. RFP (red) marks wild-type PRs (-/+) and yellow dotted lines encircle *ppr* ^*A*^ mutant PRs (-/-, lacking RFP). Rhabdomeres are costained with Phalloidin/Actin (blue). Flies were raised in constant dark and dissected and fixed under dim red light. Prior to fixation, flies were kept in dark (H) or blue light (I) for 1.5 min, allowing Arr2::GFP to translocate to rhabdomeres. Alternatively, flies were kept in blue light for 30 min and then shifted to orange light for 60 min to assess release of Arr2::GFP from rhabdomeres (J). (K) Quantification of the difference in green fluorescence intensity between mosaic ommatidia of *ppr* and wild-type rhabdomeres. Error bars represent ± SEM; statistical significance was determined using a two-tailed Student’s *t* test (*p*-values: * <0.05, ***<0.001).

The observed transient depolarization phenotype upon repetitive stimulation, as observed in *ppr* mutant PRs (Fig 3A and 3B), could be due to impaired PLC activity [27] and/or the inability to quickly regenerate PI(4,5)P_2_ [28,42], resulting in diminished TRP activity. However, we did not find any evidence for a loss of PLC activity in *ppr* mutants. Indeed, PLC loss typically impairs PI(4,5)P_2_ hydrolysis [43] and causes a delay in repolarization [44], neither of which was observed in *ppr* mutants (Fig 3A and S3A–S3C Fig). To assess if the expression of other proteins required for the phototransduction pathway are affected in *ppr* mutant PRs, we performed western blots of many key players in the process [15,16,35]. As shown in Fig 4A, none of the proteins tested display altered expression levels in *ppr* mutant eyes (2–3-d-old, reared in the dark). Hence, *ppr* does not seem to affect proteins known to be required for the light transduction pathway. In addition, there are no hints of morphological changes in these PRs prior to testing (Fig 4B and 4C). These data indicate that PLC activity is not impaired and that most known players are present.

### Light Induces Rh1 Accumulation in *ppr* Mutant PRs

Upon photoisomerization of Rh1 to MRh1 by a photon of blue light, the latter is quickly inactivated by Arrestin2 (Arr2) binding (Fig 3C and S4E Fig) [45,46]. Subsequently, MRh1 is reisomerized to Rh1 by a photon of orange light (~580 nm), leading to the release of Arr2 [32,45]. The mechanism of Rh1 recycling requires Ca^2+^ influx through TRP channels [32–34,45,47–49]. A small fraction of Rh1/Arr2 complex is endocytosed and degraded [50]. A reduced Ca^2+^ influx results in increased levels of the Arr2/Rh1 complex, causing excessive endocytosis of Rh1, which is toxic to cells as it stresses the endolysosomal system [47,50–54].

The inability to maintain a sustained light response in *ppr* mutant eyes (Fig 3A) suggests an impaired Ca^2+^ influx in PRs [37–40], which in turn may affect the Rh1 cycle and hence lead to an increased internalization of Arr2-bound Rh1 upon exposure to light. Inducing a constitutive Ca^2+^ influx, however, severely impairs the function and affects the morphology of *ppr* mutant PRs, even in newly eclosed flies (S3D and S3E Fig), possibly due to synergizing effects of Ca^2+^ toxicity and mitochondrial stress. To assess whether Rh1 internalization is affected, we performed whole mount antibody staining for Rh1 [47,55]. As shown in Fig 4D–4G, *ppr* mutants show no defect in the dark but exhibit Rh1 accumulation when exposed to light. Note that although Rh1 is found throughout the rhabdomeres when sections are performed (S4A–S4D Fig), in whole mount preparations Rh1 is detected on the outer rim as the antibodies cannot penetrate the membrane stack. However, the whole mount protocol reveals internalized Rh1 much better (Fig 4D–4G) than stained sections (S4D Fig) [47,55–57]. In addition, brief exposures to blue light followed by orange light cause very similar accumulations of Rh1 in the cytoplasm, indicating a defect in Rh1 cycling in *ppr* mutant PRs (S4E Fig). Since increased cytoplasmic Rh1 is known to cause degeneration of PRs [52], our data suggest that Rh1 mediates degeneration of *ppr* mutant PRs.

To determine if increased Rh1 internalization in *ppr* mutant PRs is associated with a defect in Arr2 dynamics, we tested Arr2 translocation to rhabdomeres upon blue light exposure and its release following orange light exposure. To detect Arr2, we expressed Arr2::GFP under the control of the *Rh1* promoter (Green or Gray, Fig 4H–4J), which is active in R1–R6 PRs [31]. We generated small *ppr* mutant mitotic clones with *ey*-FLP in otherwise heterozygous retina. The mutant *ppr* PRs (dotted circles, Fig 4H–4J) can be distinguished from wild-type PRs by the absence of RFP (shown in red). Upon blue light exposure, Arr2 translocates to the rhabdomere membranes (binding to Rh1). The subsequent exposure to orange light relocates Arr2 to the cytoplasm as it is released from Rh1 [31,45,52]. In wild-type and *ppr* mutant clones, Arr2::GFP levels are low in rhabdomeres when flies are kept in the dark (Fig 4H). However, upon exposure to ~1.5 min of blue light, Arr::GFP levels are increased in wild-type as well as *ppr* mutant rhabdomeres (Fig 4I). To assess the release of Arr2::GFP from rhabdomeres, we kept flies in blue light for 30 min followed by a 60 min exposure to orange light prior to fixation. As shown in Fig 4J and 4K, we observe a higher level of GFP florescence in *ppr* mutant rhabdomeres than in wild-type rhabdomeres, indicating the slow release of Arr2 in *ppr* mutant rhabdomeres. Moreover, light-induced internalized Rh1 in *ppr* mutant PRs colocalizes with Arr::GFP punctae when compared to wild-type PRs (S4F Fig). Hence, our data indicate that impaired dynamics of Arr2 release from Rh1 in *ppr* mutant PRs leads to increased internalization of Rh1 and toxicity.

### PR Degeneration in *ppr* Is Mediated by Rh1

**Fig 5:**
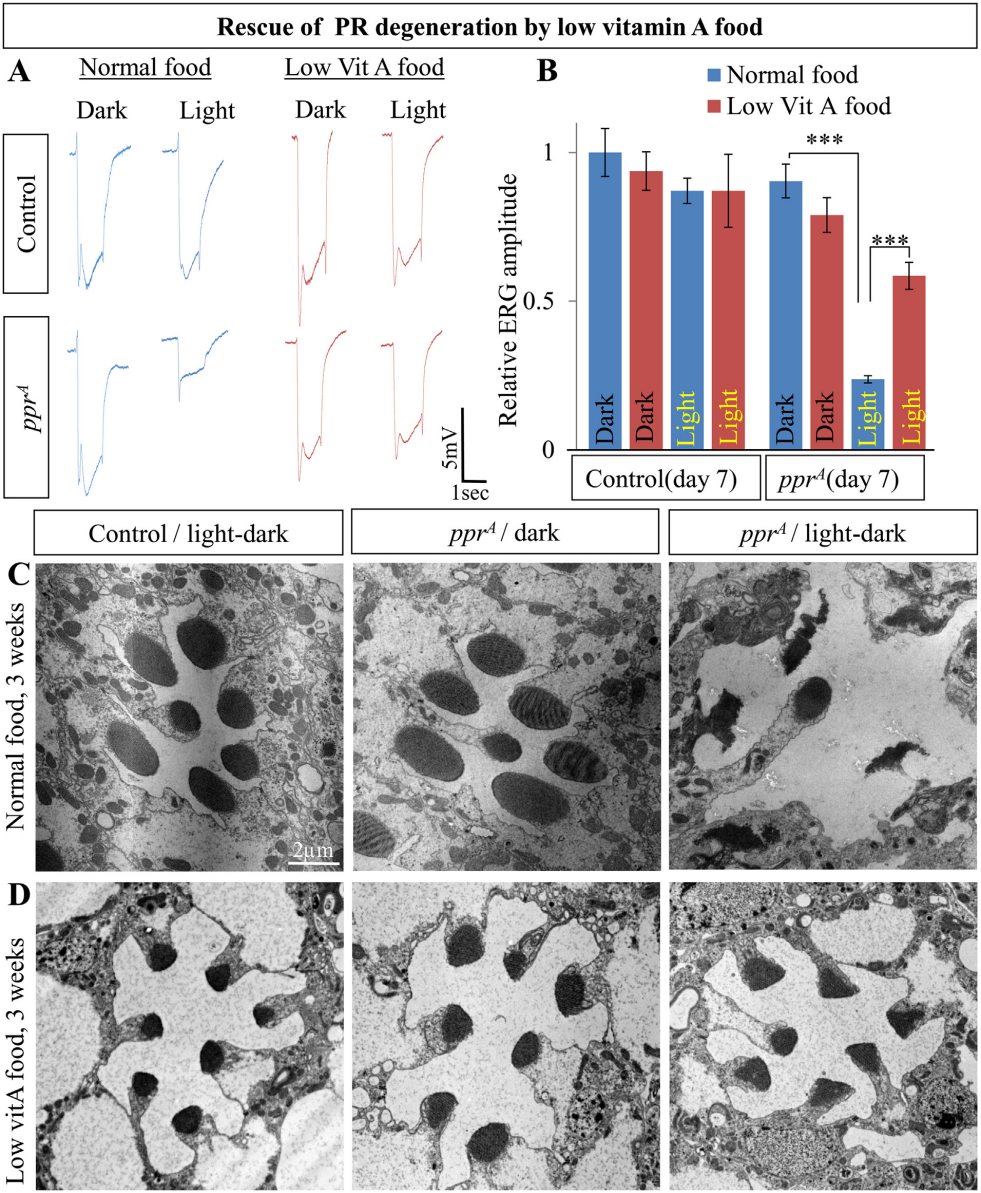
Rh1-dependent degeneration of *ppr* mutant PRs. (A) ERG traces from control (top) or *ppr* ^*A*^ (bottom) eye clones. Flies were raised on normal food (blue, left) or low vitamin A food (red, right) and kept in the dark or constant light for seven days. (B) Quantification of relative ERG amplitude from the experiment shown in A. Error bars represent ± SEM; two-tailed Student's *t* test (*p*-value ***<0.001). (C, D) TEM of control and *ppr* ^*A*^ ommatidia. Flies were raised on normal food (C) or low vitamin A food (D) and raised in the dark (middle, *ppr* ^*A*^) or 12 h light/dark cycle (left, control and right, *ppr* ^*A*^) for three weeks.

To test if excessive Rh1 internalization causes PR degeneration in *ppr* mutant eyes, we examined whether reducing Rh1 suppresses the light-dependent degeneration of *ppr* PRs. Maturation of Rh1 requires the binding of the chromophore, 11-cis 3-hydroxyretinal, to the opsin moiety. In the absence of the chromophore, opsin is not exported to the rhabdomere but is instead degraded [17,58,59]. In flies, the major source of the chromophore is derived from dietary *β*-carotene/vitamin A [59]. Indeed, Rh1 levels can be reduced to less than 3% by raising flies in vitamin A-deficient food, and this reduction has been shown to suppress Rh1-mediated PR degeneration [47,56]. Interestingly, under constant light or dark conditions, the ERG amplitude in flies deprived of *β*-carotene is comparable to those raised on normal food (Fig 5A and S5A Fig). *ppr* mutants raised in constant light for seven days on normal food display ERG amplitude that is ~ 20% of control (Fig 5A and 5B), whereas *ppr* mutants raised on vitamin A-deficient food display an ERG amplitude that is ~60% of control. Hence, removal of most Rh1 in PRs (S5A Fig) strongly suppresses the neurodegenerative phenotypes associated with the loss of *ppr*.

To assess whether depriving flies of vitamin A suppresses the morphological alterations of *ppr* mutant PRs induced by light exposure, we performed TEM of the retina in flies reared in a 12 h light/dark cycle for three weeks. As previously shown [52], rhabdomeres of flies deprived of vitamin A are small, since Rh1 is an important structural component of rhabdomeres (Fig 5D). When raised on normal food, the morphology of *ppr* mutant rhabdomeres is severely affected (Fig 5C), but PRs of mutant flies deprived of vitamin A are indistinguishable from controls, albeit reduced in size in both cases (Fig 5D and S5B Fig). Combined with the Arr2 data, these results indicate that increased Rh1 internalization is a major cause of PR degeneration in the absence of *ppr*.

We have previously shown that the retromer complex alleviates endolysosomal stress in PRs by preventing Rh1 from entering the endolysosomal pathway. Hence, overexpression of subunits of the retromer promotes its activity and suppresses Rh1-induced endolysosomal trafficking defects in some mutants [56]. Similarly, overexpression of *vps35* in PRs suppresses or delays the neurodegenerative defects in *ppr* mutants (S5C Fig). These data provide further support that aberrant Rh1 internalization/degradation is a major cause of PR degeneration in the absence of *ppr*.

### Mitochondrial-Derived Transcript Levels Are Reduced in *ppr* Mutants

**Fig 6:**
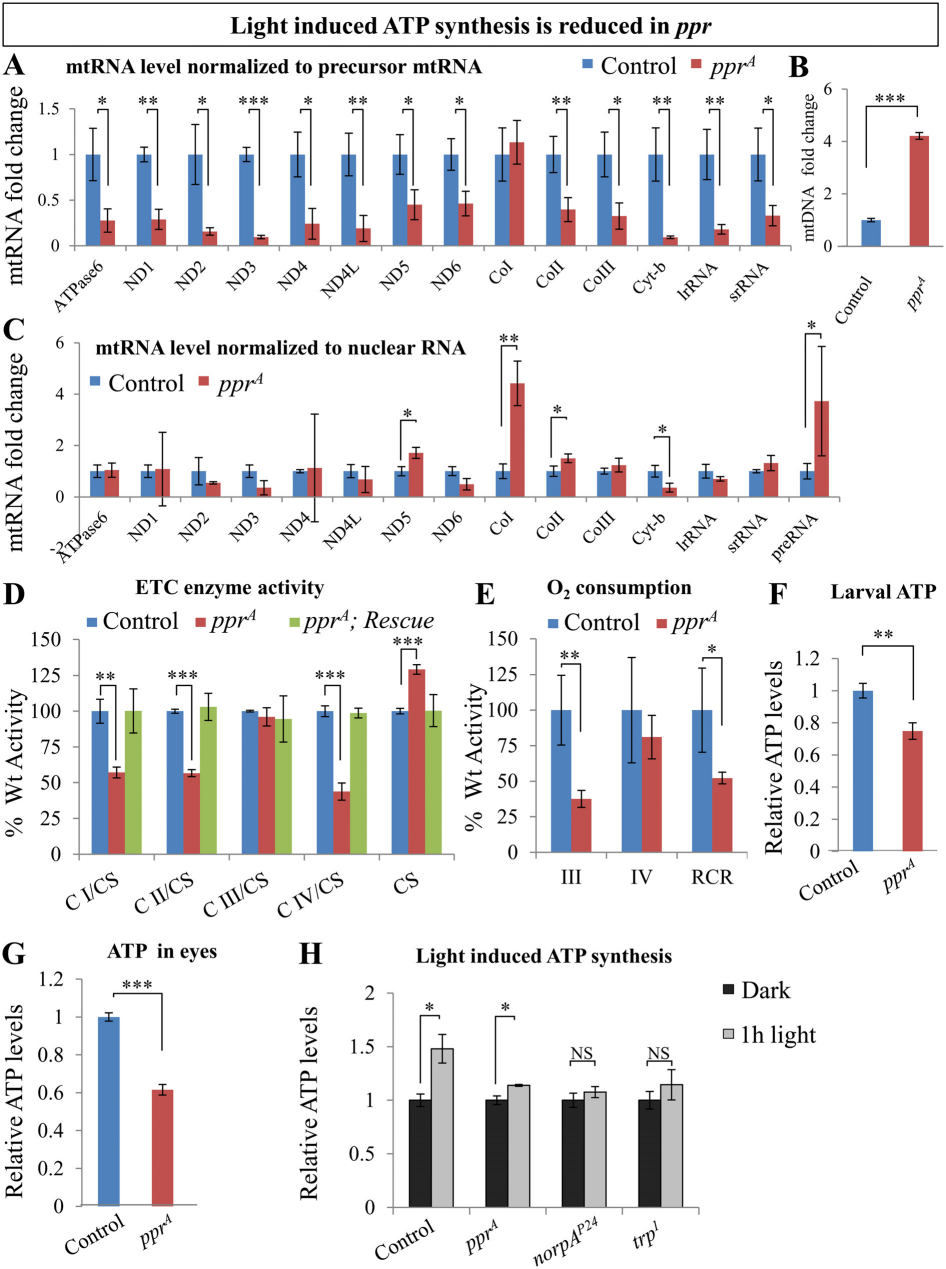
Light-induced ATP synthesis is reduced in *ppr* mutant eyes. (A) Relative mtRNA levels, normalized to precursor RNA transcribed from the heavy strand (+) of the mitochondrial genome, in control and *ppr* ^*A*^ third instar larvae. (B) Relative mtDNA content, normalized to nuclear DNA, in control and *ppr* ^*A*^ third instar larvae. (C) Relative mtRNA levels, normalized to nuclear RNA RP49. (D) Activities of mitochondrial electron transport chain (ETC) protein complexes (CI, CII, CIII, CIV) and Citrate synthase (CS) from third instar larval extracts. Genotypes shown are control, *ppr* ^*A*^ and *ppr* ^*A*^; genomic rescue (CH322-75O21). ETC complex activity was normalized to CS, and data are expressed as percentage of the activity detected in controls. (E) O_**2**_ consumption assayed by polarography. O_**2**_ consumption was measured from isolated third instar larvae-derived mitochondria in the presence of CI-specific substrates. State III is the ADP-stimulated O_**2**_ consumption rate; State IV represents the ADP-limited O_**2**_ consumption rate; RCR is the Respiratory Control Ratio (state III rate / state IV rate). (F–G) Relative ATP levels from control and *ppr* ^*A*^ third instar larval extracts (F) and adult eyes (exposed to 1 h light, 1,800 Lux) (G). (H) Relative change in ATP levels in adult heads upon 1 h exposure to light (1,800 Lux). In A–H, error bars represent mean ± standard deviation (SD); statistical significance was determined using a two-tailed Student’s *t* test (*p*-values: *** < 0.001, ** < 0.01, * <0.05).

Since Ppr is a mitochondrial protein, and the phototransduction process consumes a significant amount of ATP [5,60,61], we sought to assess whether loss of *ppr* compromises ATP production. LRPPRC, the human homolog of *ppr*, and its homologs are required for polyadenylation and stability of mitochondrial RNA (mtRNA) and translation [62,63]. Mitochondrial DNA is transcribed as two long polycistronic precursor RNAs [64]. The precursor RNAs are then processed to create smaller mtRNAs, which are stabilized by the addition of a polyA tail [64,65]. To assess whether *ppr* is required for mtRNA stability, we quantified the mtRNA levels for 14 transcripts by RT-qPCR and normalized this data to mitochondrial precursor RNA. As shown in Fig 6A, except for Complex I, all mtRNA levels are significantly reduced in mitochondria of *ppr* mutant larvae, in agreement with a role of Ppr proteins in mtRNA stability [62,63,66–69]. Moreover, the mtDNA content, normalized to nuclear DNA, in *ppr* mutants is about four times higher than in control larvae (Fig 6B), suggesting that the loss of mtRNA may induce a compensatory increase in mitochondrial biogenesis. Indeed, when we normalize the mtRNA levels with nuclear RNA (RP49), we found an increase in mitochondrial precursor RNA levels (right of Fig 6C) consistent with an increase in mitochondrial biogenesis (Fig 6B). However, normalization of processed mtRNA with nuclear RNA reveals that the mtRNA of ND5, CoI, and CoII are up-regulated and Cyt-b is down-regulated, whereas others are unchanged (Fig 6C). Hence, the overall mtRNA levels in a cell are not dramatically altered. These data suggest the presence of a compensatory response, which induces mitochondrial biogenesis in *ppr* mutant and can, in part, counterbalance the reduced mtRNA stability per mitochondrion.

### Loss of *ppr* Leads to Decreased Mitochondrial ATP Production

Given that mtRNAs encode 13 different proteins that are all components of the mitochondrial electron transport chain (ETC) complex (I, III, IV, and V) [13,64], we sought to determine enzymatic activities of individual ETC components from whole cell lysates (Fig 6D) or isolated mitochondria (S1 Data). We also measured Citrate synthase (CS) activity to normalize ETC complex activity. We observed significant decreases in the activities of Complex I, Complex II, and Complex IV in *ppr* mutant larvae (Fig 6D). The decreased activity of Complex II is striking, as its subunits are encoded in the nucleus [70]. Nevertheless, these data are consistent with the reduced Complex II activity that was observed in *LRPPRC* knockout mice [63]. Finally, the defects in ETC activity are rescued by a wild-type genomic copy of *ppr*, showing that the loss of *ppr* is indeed responsible for these phenotypes.

To assess mitochondrial energy production, we measured the rate of oxygen consumption of intact mitochondria in vitro by polarography. In the presence of the Complex I-specific oxidizable substrates malate and glutamate, *ppr* mutant mitochondria exhibit a significant defect in state III (ADP-stimulated O_2_ consumption rate), resulting in a decreased respiratory control ratio (RCR), defined as the ratio of state III to state IV (ADP-limiting O_2_ consumption rate) (Fig 6E). The observed partial deficiencies of several ETC complexes in *ppr* mutants, combined with the defective respiration of isolated *ppr* mutant mitochondria (manifesting as reduced state III rate and RCR), are indicative of a reduced efficiency of oxidative phosphorylation (OXPHOS), or in other words, reduced OXPHOS-dependent ATP production [71]. We therefore measured steady state levels of ATP and observed reduced ATP levels in *ppr* mutant larvae when compared to control animals (Fig 6F). Together, these results provide compelling evidence that *ppr* regulates mitochondrial RNA levels and thereby affects OXPHOS and ATP levels.

PRs are known to consume up to 10% of total ATP in blowflies [60,72]. ATP consumption increases 5-fold above baseline in *Drosophila* PR in the presence of light [5,6]. Thus, we tested ATP levels in *ppr* mutant eyes exposed to light for 1 h (Fig 6G) and found that the ATP deficit in mutant PRs is about twice as high (40%) as in the third instar larvae (20%). Furthermore, we investigated the change in ATP levels following light exposure in control and *ppr* mutant heads. Interestingly, there is a significant increase (48%) in ATP levels in wild type controls upon a 1 h light exposure but only a subtle increase (13%) in ATP level in *ppr* mutants (Fig 6H). In summary, there is impaired ATP production in the eye of *ppr* mutants.

It has been shown that mitochondrial activity is triggered by Ca^2+^ influx in neurons [72–75]. We therefore measured changes in ATP following light exposure in mutants that have an impaired Ca^2+^ influx (*trp* [38] and *norpA/PLC* [37,76]). Indeed, these mutants fail to increase ATP levels following light exposure (Fig 6H), suggesting that a Ca^2+^ influx is required to activate mitochondrial ATP production. These data indicate the presence of a feedback mechanism required for ATP generation to ensure the continuity of the phototransduction process.

### Bicoid Stability Factor, Another Pentatricopetide Repeats-Containing Protein Plays a Partially Redundant Role with Ppr

**Fig 7:**
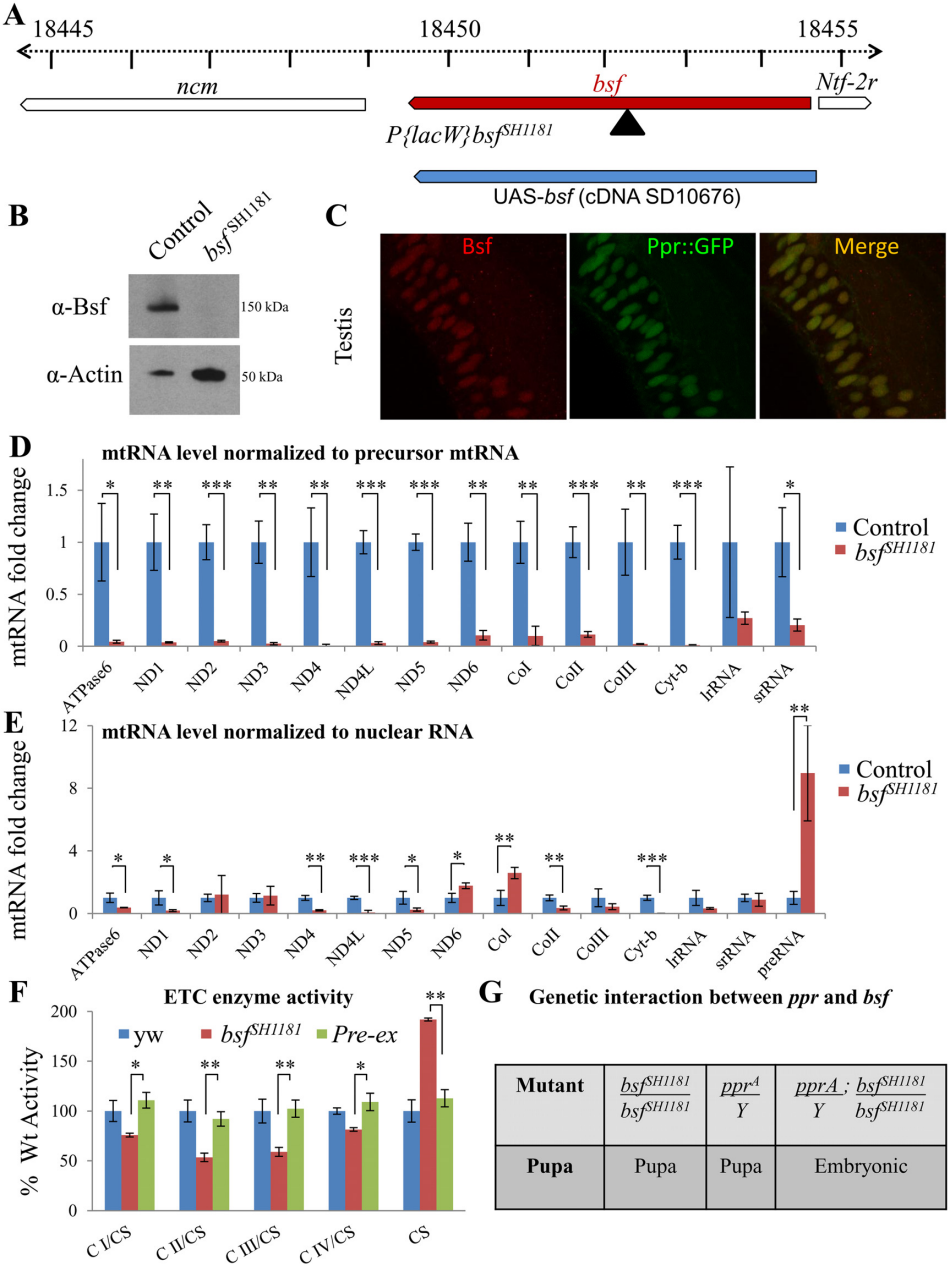
*ppr* and *bsf* are partially redundant genes. (A) In the *bsf* ^*SH1181*^ allele, P{lacW} is inserted within the coding sequence of *bsf*. (B) No Bsf protein is detected on western blots of larval extract of *bsf* ^*SH118*^. (C) Colocalization of Bsf (red) with GFP-tagged Ppr (green) in adult testes. (D, E) Relative mtRNA levels from control (precise excision line) and *bsf* ^*SH1181*^ third instar larvae normalized to mitochondrial precursor RNA (D) and nuclear RNA RP49 (E). (F) Activities of mitochondrial ETC protein complexes (CI, CII, CIII, CIV) and CS from *yw*, *bsf* ^*SH1181*^ and precise excision (Pre-Ex). ETC complex activity was normalized to CS, and data are expressed as percentage of the activity detected in controls. In D–F, error bars represent mean ± SD; statistical significance was determined using a two-tailed Student’s *t* test (*p*-values: *** < 0.001, ** < 0.01, * <0.05). (G) Lethal stages of *bsf* ^*SH1181*^, *ppr* ^*A*^, and double mutants (*ppr* ^*A*^; *bsf* ^*SH1181*^).

Besides *ppr*, the *Drosophila* genome contains a single other gene that contains PPR motifs, *bicoid stability factor* (*bsf*) [66]. RNAi-mediated knockdown of *bsf* also affects mtRNA stability [66]. Hence, *ppr* and *bsf* may be partially redundant. We identified an allele of *bsf* (*bsf* ^*SH1181*^; Fig 7A) that appears to be a null allele, as no Bsf protein was detected in western blots (Fig 7B). Ubiquitous expression of *bsf* cDNA rescues the pupal lethality associated with *bsf* ^*SH118*^. Finally, Bsf also colocalizes with Ppr::GFP (Fig 7C). These data permitted us to compare and contrast the mitochondrial phenotype of *ppr* mutants to *bsf* mutants. When we assessed mtRNA levels, as shown in Fig 7D and 7E, *bsf* mutants show a similar phenotypic profile to *ppr* mutants although typically more severe. In addition, *bsf* mutants also show defects in the ETC activity (Fig 7F). Similar to *ppr* mutants, CII activity is reduced in *bsf* mutants. However, unlike *ppr*, *bsf* mutants display a severe reduction in CIII activity. These data suggest that Ppr and Bsf may play partially redundant functions. To test this hypothesis, we created double mutants. As mentioned before, *ppr* and *bsf* mutants cause pupal lethality. However, “*ppr*–*bsf*” double mutants die as embryos (Fig 7G), suggesting that Ppr and Bsf are partially redundant.

### Loss of *ppr* Does Not Cause Oxidative Stress

**Fig 8:**
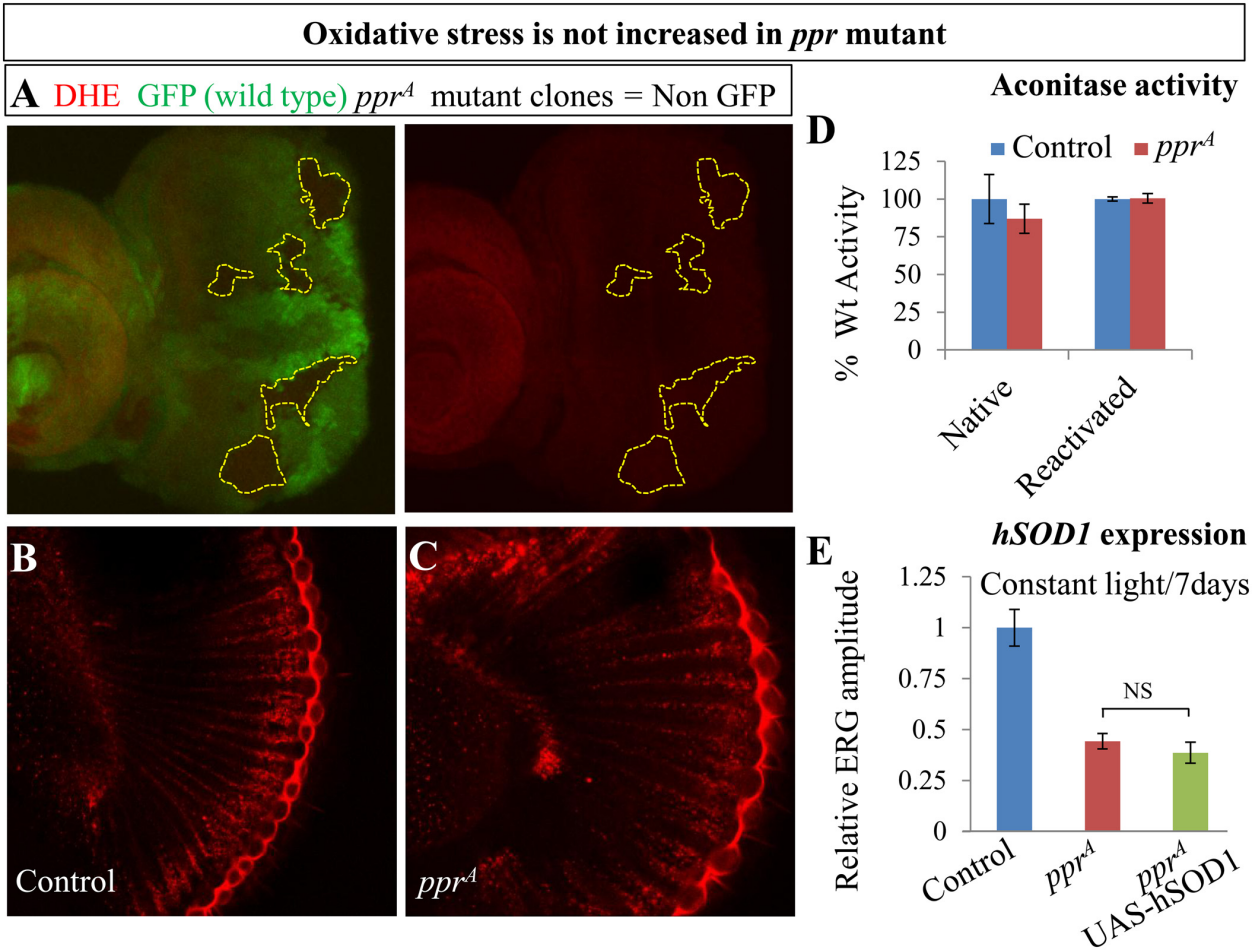
PR degeneration is not induced by oxidative stress in *ppr* mutants. (A–C) Detection of ROS levels by DHE (red) staining in *ppr* mutant clones in eye imaginal discs. Mutant clones, encircled by dotted lines, are marked by loss of GFP (green). (B, C) Detection of ROS levels by DHE staining (red) in control (B) and *ppr* mutant (C) eyes from adult flies exposed to 24 h light (1,800 Lux). (D) Aconitase activity, which is negatively correlated to ROS levels because of its sensitivity to oxidation, is measured in mitochondrial extracts from third instar larvae (Native) or upon treatment with a reducing agent (Reactivated) to control for variations in the total amount of enzyme. Error bars represent ± SD. (E) Relative ERG amplitude from retinas of control, *ppr* ^*A*^ and *ppr* ^*A*^ expressing hSOD1 in R1-R6 using Rh1-Gal4. All flies carried Rh1-GAL4 in this experiment. Flies were raised in constant light for seven days. Error bars represent ± SEM; NS (two-tailed Student’s *t* test not significant).

Mitochondrial defects have been shown to cause elevated ROS levels and retinal degeneration in mammals and flies [9–13,77]. We therefore tested if ROS levels are elevated in *ppr* mutants by staining with dihydroethidium (DHE), a dye which detects superoxide radicals [78,79]. As shown in Fig 8A, *ppr* mutant clones in eye imaginal discs, marked by loss of GFP, do not show differences in fluorescence intensity when compared to neighboring wild-type tissue. We also performed DHE staining in adult eyes exposed to 24 h constant light. As shown in Fig 8A, the level of DHE staining in mutant eye is similar to control eye (Fig 8B–8C). We also assessed ROS levels by assaying mitochondrial aconitase activity. The native activity of this enzyme is extremely sensitive to elevated ROS [80] and a highly reliable readout in *Drosophila* [11,77]. As shown in Fig 8D, aconitase activity in mutant animals is comparable to control, suggesting that ROS levels are not affected in *ppr* mutants. Furthermore, we overexpressed human copper-zinc superoxide dismutase (hSOD1), a potent suppressor of neurodegeneration induced by ROS in flies [12,77,81], in *ppr* mutant PRs. However, we did not observe a suppression of the degenerative phenotype (Fig 8E), again implying that PR degeneration in *ppr* mutants is not induced by oxidative stress.

### Mutations That Cause a Loss of CS or Pyruvate Dehydrogenase Also Lead to Light-Induced PR Degeneration

**Fig 9:**
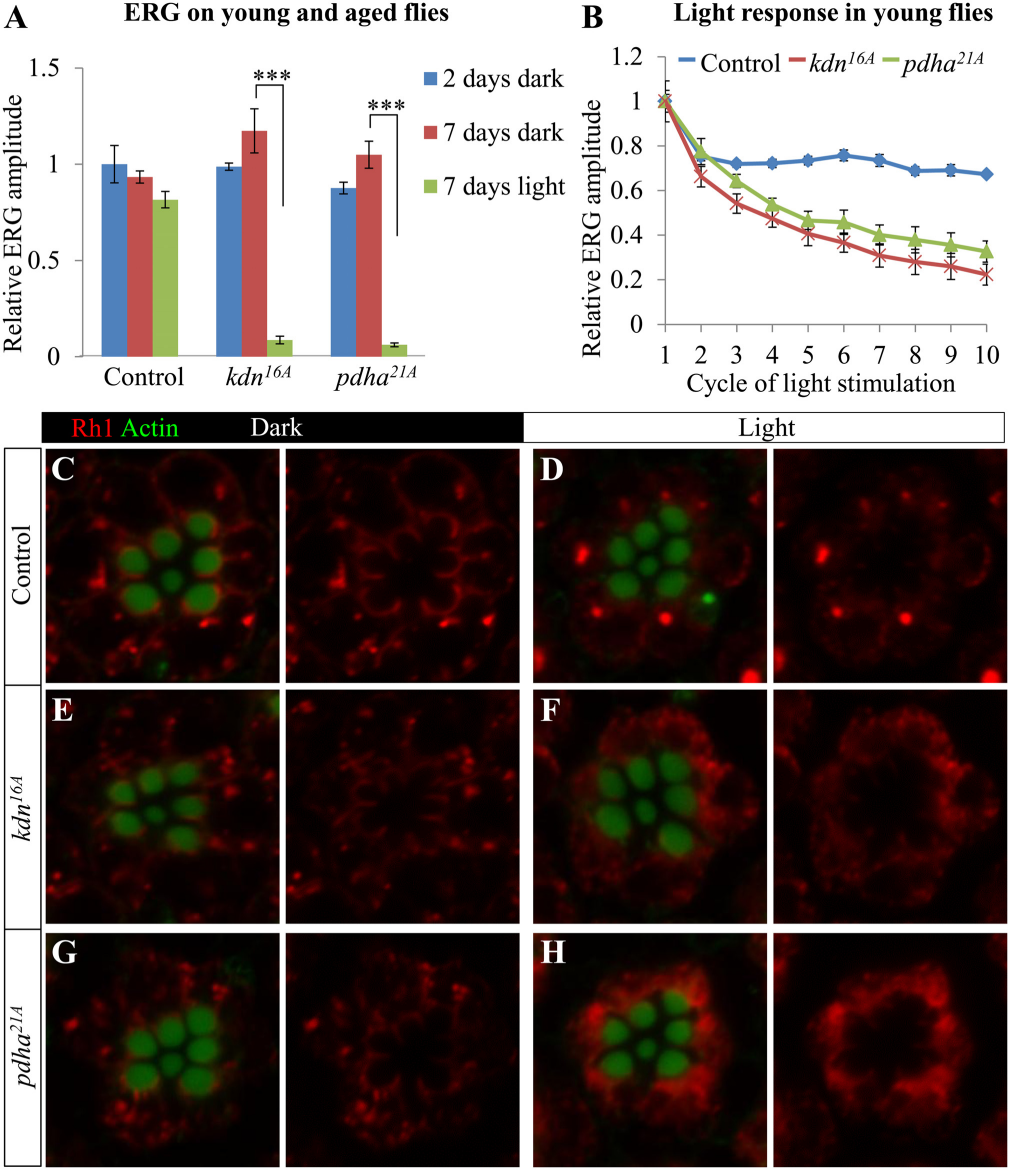
Light-induced degeneration due to loss of CS and Pyruvate dehydrogenase. (A) Quantification of relative ERG amplitudes from control, *kdn* ^*16A*^, and *pdha* ^*21A*^ eye clones. Flies were reared in the dark for two (blue) or seven days (red) or in constant light for seven days (green). (B) Quantification of relative ERG amplitudes measured during repetitive light stimuli (1 sec light and 1.5 sec dark, as shown in Fig 3A) from control, *kdn* ^*16A*^, and *pdha* ^*21A*^ eye clones. Error bars represent ± SEM; Student's *t* test (*p*-value: ***<0.001). (C–H) Whole mount Rh1 (red) immunostaining in control, *kdn* ^*16A*^, and *pdha* ^*21A*^ mutant PRs. Rhabdomeres are marked by Phalloidin and Actin (green). The flies used in this experiment were 2–3 d old and reared in the dark (C, E, G) or exposed to ~24 h of light (D, F, H).

Based on our findings, loss of *ppr* causes reduced ATP production but does not alter steady state ROS levels. However, *ppr* deficiency causes a severe loss of ERG responses and Rh1 accumulation upon repetitive light stimulation as well as a progressive light-induced PR degeneration. In the genetic screen that permitted the isolation of *ppr*, we identified mutations in numerous genes whose proteins are targeted to mitochondria [20]. To assess if mutations in genes that have been shown to affect ATP production display similar phenotypes, we evaluated an embryonic lethal allele of *knockdown* (*knd* ^*16A*^) [21], which encodes a homolog of CS, and *CG7010* (*pdha* ^*21A*^, G170E) [20], which encodes the E1 subunit of Pyruvate dehydrogenase. Loss of CS impairs the tricarboxylic acid (TCA) cycle and hence NADH and ATP production [82,83], whereas Pyruvate dehydrogenase converts pyruvate to acetyl-CoA and mediates entry of glycolytic products into the TCA cycle [84]. Mutant clones in the eyes of *knd* and *pdha* show normal primary ERG amplitudes in young flies and flies aged in complete darkness, similar to *ppr* mutant PRs (Fig 9A). However, a seven-day exposure to light nearly abolishes ERG amplitudes in these mutants, whereas wild type control PRs are barely affected. Hence, loss of *kdn* or *pdha* causes a severe light-induced degeneration. In addition, both mutants fail to sustain the ERG amplitude upon repetitive stimulation in young animals (Fig 9B), similar to the phenotypes associated with the loss of *ppr* (Fig 3A). These observations suggest that perturbations of oxidative metabolism leading to loss of ATP production in both mutants underlie these phenotypes.

Given that loss of *ppr* induces Rh1 accumulation upon light exposure, we tested Rh1-localization in *knd* and *pdha* mutant PR of flies raised in the dark. Rh1 localization is indistinguishable from controls in 2-d-old flies (Fig 9C, 9E, and 9G). Similar to *ppr* mutant PRs (Fig 4G), an ~24 h exposure to constant light leads to a substantial increase in cytoplasmic Rh1 in *knd* and *pdha* mutants when compared to controls (Fig 9D, 9F, and 9H). Finally, as shown in S6A and S6B Fig, mutant clones of *knd* and *pdha* in eye discs do not show any change in DHE staining, suggesting that loss of these enzymes does not affect ROS production. In summary, the key phenotypes associated with the loss of *ppr* in the eye are very similar to those of *knd* and *pdha*, suggesting a common underlying pathology.

### PR Degeneration in *sicily* Is Enhanced by Exposure to Light

Since increased ROS is commonly associated with mitochondrial dysfunction and causes retinal degeneration in flies and humans [9–11,13,77], we tested whether mutations that severely affect the ETC and exhibit a severe increase in ROS levels cause both a light-dependent and light-independent degeneration. Mutations in *sicily* show a severe reduction in Complex I activity, a reduction in ATP levels (S7A Fig), and a significant increase in ROS production and PR degeneration [11,77]. In dark-reared young flies, ERG amplitudes recorded from *sicily* mutants are comparable to controls (S7B Fig). When raised in the dark for seven days, *sicily* mutant eyes exhibit a ~50% reduction in ERG amplitude, whereas the ERG amplitudes of *sicily* mutant eyes is reduced by ~80% when the flies are kept in constant light for seven days (S7B Fig). These findings suggest that both light-independent and light-dependent mechanisms cause degeneration in *sicily* mutant PRs.

As noted in *ppr*, *kdn*, and *pdha* mutants (Figs 3A and 9B), *sicily* mutants also show a loss of ERG amplitude upon repetitive stimulation in young animals (S7C Fig). Upon exposure to light for 7 d, we observe an increase in Rh1 levels in the cytoplasm of *sicily* mutant PRs (S7E Fig) when compared to controls (Fig 9D). Finally, Rh1 localization in dark-reared *sicily* mutant PRs is indistinguishable from controls (Fig 9C and 9D), similar to what we observed in *ppr*, *kdn*, and *pdha* mutant PRs (Figs 4 and 9). These results indicate that in *sicily* mutants, increased ROS levels [11,77] promote a degeneration that is exacerbated by Rh1 accumulation upon light exposure.

## Discussion

In a forward genetic screen designed to identify mutations in essential genes that cause neuronal degeneration, we identified mutations in numerous nuclear genes that encode mitochondrial proteins. One of these genes corresponds to *ppr*, a homolog of human *LRPPRC* (Fig 1). Interestingly, *ppr* mutant PRs do not degenerate in the dark, in contrast to other mitochondrial mutants such as *sicily* [11] and *sdhA* [10], suggesting that a different mechanism underlies the degeneration in *ppr* mutants. Intriguingly, unlike many other mitochondrial mutants [10–13,77], loss of *ppr* does not affect ROS levels but impairs ATP production (Figs 6G and 6H and 8A–8D), suggesting that a reduced ATP production underlies the light-dependent degeneration. This hypothesis is supported by the identification and characterization of mutations in two other genes encoding Pyruvate dehydrogenase and CS, which play an important role in the TCA cycle. Both are critical to sustain mitochondrial ATP production [82,83,85–87]. These results, however, do not rule out the possibility that the ratios of other metabolites will be altered because of the different mitochondrial defects, and that these alterations contribute to degeneration. Nevertheless, our results indicate that mutations that affect mitochondrial ATP production without altering ROS levels do not cause PR degeneration in the absence of neuronal activity. This is in sharp contrast with other mitochondrial mutations like *sdhA* that display increased ROS [10]. Hence, reduced neuronal activity in this subgroup of mitochondrial mutants has neuroprotective effects.

In a French Canadian population, mutations in human *LRPPRC* have been associated with Leigh Syndrome, an autosomal recessive neurodegenerative disorder with onset in infancy [23]. LRPPRC is a key regulator of mtRNA polyadenylation and stability as well as translation [62,63,69], and loss of *LRPPRC* causes a decrease in mtRNA abundance, defects in translation, ETC activity, and mitochondrial ATP production. In agreement with the phenotypes associated with loss of *LRPPRC*, we observe a reduction in mtRNA stability in mitochondria of *ppr* mutants (Fig 6A).

We also show that *ppr* and *bsf*, the two fly homologs of *LRPPRC*, play partially redundant roles. We find that CIII activity, which is not affected in *ppr* mutants (Fig 6D), is significantly lower in *bsf* than in *ppr* mutants (Fig 7F). In contrast, CIV activity is significantly down-regulated in *ppr* mutants (Fig 6D) when compared to *bsf* mutants (Fig 7F). Surprisingly, both *ppr* and *bsf* mutants display a decreased activity of the nuclear-encoded CII (Figs 6D and 7F), a phenotype also observed in *LRPPRC* knockout mice [63]. We do not know the cause for this reduced CII activity. Reduced CII activity in *ppr* and *bsf* mutants as well as in *LRPPRC* knockout mice may be related to the increase in mitochondrial DNA and transcription, as observed in mouse knockouts for *Mterf3* and *Tfb1m* [88,89].

Finally, we show that mitochondria isolated from *ppr* mutants show reduced ADP-stimulated oxygen consumption (Fig 6E), suggesting a defect in OXPHOS leading to reduced mitochondrial ATP production (Fig 6F–6H). In summary, features associated with the loss of *ppr* in flies are similar to what has been described in human cell and mouse experiments [62,63,68,69,90].

Phototransduction is a high ATP-consuming process, and eyes have been estimated to consume 10% of total ATP produced in blowflies [5,60,72]. Moreover, neurons primarily rely on mitochondrial OXPHOS for ATP production [72,91,92]. We show that ATP synthesis increases upon exposure to light in controls suggesting the need for a constant energy supply during phototransduction (Fig 6H). Ca^2+^ has been shown to activate ATP synthesis in mitochondria, and we observe that blocking Ca^2+^ influx in PRs also inhibits light-induced ATP production (Fig 6H). Hence, the failure to maintain PR activity during repetitive light exposure in young *ppr* animals (Fig 3A) may result from reduced mitochondrial activity.

In *ppr* mutant PRs, a defect in Rh1 cycling (S4E Fig), due to reduced Ca^2+^ influx as predicted by reduced ERG amplitude (Fig 3A), induces excessive internalization of Rh1 (Fig 4G and S4E Fig). Excessive Rh1 internalization is known to overload the endolysosomal system, resulting in neurodegeneration upon prolonged light exposure [47]. Indeed, reducing Rh1 by reducing vitamin A uptake strongly suppresses the PR degeneration associated with *ppr* mutants (Fig 5A–5D). The observation that the overexpression of the retromer complex protein Vps35, which recycles internalized Rh1 and protects PRs from degeneration [56], partially rescues light-induced ERG phenotypes in *ppr* (S5C Fig) provides further support that excessive Rh1 mediates degeneration of *ppr* mutant PRs.

Mitochondrial dysfunction is one of the leading causes of neurodegeneration [93]. However, mitochondrial disease-associated phenotypes differ significantly depending on the gene that is affected and the nature of the mutations [94]. Comparing the phenotypes observed in previously characterized *Drosophila* mitochondrial genes allows us to start subdividing them into more discrete phenotypic groups that can be correlated with the observed physiological defects. For example, *sdhA* mutants exhibit PR degeneration in the dark and an increase in ROS, yet the ATP levels remain normal [10]. In contrast, *ppr*, *kdn*, and *pydh* mutants exhibit reduced ATP levels [82,83,85–87,90], unaltered ROS levels and their PR only degenerate when exposed to light (Figs 2 and 9A). Mutations that cause reduced ATP production and increased ROS levels may show an intermediate phenotype. *sicily* mutants show a severe CI deficiency, severely increased ROS levels [11,77] and reduced ATP levels (S7A Fig). Indeed, *sicily* mutants exhibit a light-independent PR degeneration that is accelerated by light exposure (S7B Fig). Consistent with phenotypes observed in *ppr* mutants, *sicily* mutant PRs fail to sustain ERG amplitude upon repetitive light exposure (S7C Fig) and accumulate Rh1 when exposed to light (S7D Fig). These observations suggest that reduced mitochondrial ATP production exacerbates the phenotype induced by excessive ROS production through Rh1-mediated toxicity. In summary, our data suggest that the mechanisms that underlie the neurodegenerative phenotypes in a number of mitochondrial mutants are due to differences in key parameters like ATP production and ROS levels. Obviously, other mechanisms are also likely to play a role in mitochondrial dysfunction-associated neurodegeneration.

## Materials and Methods

### Fly Strains and Genetics

#### Mutagenesis and mutant strains

To isolate lethal mutations on the X chromosome, mutagenesis was performed on an isogenized fly strain *y w P{neoFRT}19A*. Mutagenesis was performed using ethylmethane sulfonate (EMS, 7.5–15 mM). Detailed mutagenesis methods for isolating *ppr*, *kdn*, *pdha*, *and sicily* alleles were described earlier [20,21]. Fly strain *bsf* ^*SH1181*^ is a lethal P-element insertion in *bsf* [95]. The original *bsf* ^*SH1181*^ line, obtained from Bloomington Stock Center, was backcrossed to wild-type for seven generations to remove second site lethal mutations. We mobilized the P-element in the *bsf* ^*SH1181*^ line and identified a viable precise excision line to use as control. Other mutant lines used in this work are *trp* ^*1*^ [38]and *norpA* ^*P24*^ [76,96].

#### Mapping

*y w mutant P{neoFRT}19A/FM7c*, *Kr-GAL4 UAS-GFP* flies were crossed to a set of large duplication lines that cover part of the X chromosome (~1–2 Mb) on the Y chromosome to roughly map the lethality and to generate rescued males. Mutants rescued by the same duplication were then crossed inter se to establish complementation groups. *ppr* alleles were rescued by *Dp(1;Y) y* ^*2*^67g19.1 (FBab0003240) that covers cytological region 2B17-2B18;20A3;h1-h25B. Through array comparative genomic hybridization (CGH), we molecularly mapped this duplication to chromosomal location *X*:184,000…1,836,000 [21]. We then performed complementation tests of *ppr* alleles with deficiencies within this region and found that *Df(1)Exel6227* (*FBab0037794*) [97] and *Df(1)BSC719* (FBab0045788) [98] failed to complement *ppr* alleles. These deficiencies share a 25 kb interval (S1 Fig). We Sanger sequenced the open reading frames in this 25 kb region and identified mutations in *CG14786* in all *ppr* alleles (Fig 1B).

#### Mutant eye clones

To generate homozygous mutant clones in the eyes of a heterozygous animal, we crossed *P{GMR-hid}SS1*, *y* ^*1*^ w* P{neoFRT}19A, *l(1)CL1 / FM7c*, *Kr-GAL4 UAS-GFP; ey-FLP(II)* females with *y w mutant P{neoFRT}19A/ Dp(1;Y)* males. *P{GMR-hid}SS1*, *y* ^*1*^ w* P{neoFRT}19A, *l(1)CL1* flies were obtained from the Bloomington stock center (FBst0005249). This technique allowed us to generate a full eye mutant clone [99]. To generate homozygous mutant clones that are marked by the absence of GFP, we crossed y w mutant* P{neoFRT}19A/ FM7c, *KrGAL4 UAS-GFP* with y w hsFLP Ubi-GFP *P{neoFRT}19A* males (a gift from David Bilder). To induce mitotic clones, first instar larvae were incubated at 37^°^C in a water bath for an hour. To generate homozygous mutant PR clones that are marked by the absence of RFP, we crossed *y w ppr* ^*A*^ *P{neoFRT}19A/ FM7c*, *Kr-GAL4 UAS-GFP* with *y w P3-RFP FRT19A;ey-FLP/SM1*. To reveal Arr2::GFP levels in homozygous mutant PR clones, marked by absence of RFP, we crossed *y w ppr* ^*A*^ *P{neoFRT}19A/ FM7c*, *Kr-GAL4 UAS-GFP* with *y w P3-RFP FRT19A; NinaE-Arr2*::*GFP*, *ey-FLP/SM1* [100]. To overexpress *vps35* [56] or *hSOD1* [81], we crossed *P{GMR-hid}SS1*, *y* ^*1*^ w* P{neoFRT}19A, *l(1)CL1 / FM7c*, *Kr-GAL4 UAS-GFP; ey-FLP(II)*Rh1-Gal4 females with *y w ppr* ^*A*^ *P{neoFRT}19A/ Dp(1;Y) y* ^*2*^ *67*g19.1; UAS-*vps35* or *y w mutant P{neoFRT}19A/ Dp(1;Y) y* ^*2*^ *67*g19.1; UAS-*hSOD1* males.

#### Low vitamin A medium

Vitamin A-deficient medium contained 10% dry yeast, 10% sucrose, 0.02% cholesterol, and 1% agar in water [56].

### Generation of Transgenic Lines

#### Genomic rescue

A 17 kb genomic BAC clone that covers *CG14786* (*ppr*), P[acman] BAC CH322-75O21, which is suitable for transgenesis in *Drosophila* [22], was used for transgenesis in fly line *y w; PBac[y* ^+^ *attP]VK00005* (FBst0009725) [101].

#### GFP tagging in *CG14786 (ppr)* genomic fragment

For a 5 kb genomic rescue construct that covers only *CG14786* (*X*:1349976…1354925), the *ppr* open reading frame was amplified from P[acman] BAC CH322-75O21 using a primer set GGGAATTCGGAATATCTGCCG ATGGTTA TC (forward) and ATAAGAATGCGGCCGCCGACGCCGCTTGGCCAGATCC (reverse). This fragment was cloned into p[w^+^]attB [102] using EcoRI and NotI sites. Transgenesis was done in fly line y w; PBac[y^+^ attP] VK00005 (FBst0009725) (FBst0009725). A BamH1 restriction site was added before the stop codon in the CG14786 5 kb genomic rescue construct by chimeric PCR. PCR amplified EGFP sequence was cloned in this BamH1 restriction site to tag Ppr at the C’ end. Transgenesis was done in fly line *y w*; PBac[y^+^ attP] VK00005 (FBst0009725) (FBst0009725) and *y w*; PBac[y^+^ attP] VK00037 (FBst0009752).

#### *CG14786 (ppr)* cDNA

The coding sequence of *CG14786* was amplified from cDNA clone UT01249 (FBcl0292583) (forward primer GGAATTCGCCACCATGCAGCGAGCACGAC TGTTG and reverse primer AAATATGCGGCCGCCTATCTAACGTGGGCG CGCAG) and cloned in the *pUASattB* vector [103] between EcoR1 and Not1 sites. Kozak consensus sequences, GCCACC, were added to the 5′. Transgenesis was done in fly line *y w; PBac[y[+]-attP]VK00037* (FBst0009752).

#### *bsf* cDNA

The coding sequence of *bsf* was amplified from cDNA clone SD10676 (FBcl0290815) (forward primer GCCACCATGGCATCCATCCTGAGGAC and reverse primer GCTCTAGACTAGGCCTTGCTGTCCGGTTTG) and cloned in the pUASattB vector [103] between Not1 and Xba1 sites. Kozak consensus sequences, GCCACC, were added to the 5′. Transgenesis was done in fly line y w; PBac[y[+]-attP]VK00033 (FBst0009750).

### ERGs

For ERG recordings, flies were immobilized on a glass slide with glue. A sharp glass-recording electrode, filled with 100 mM NaCl was placed on the surface of the eye, and another sharp glass reference electrode was inserted in the thorax. Field potential recordings were performed after three to four minutes of darkness. The PR response was digitized and recorded using AXON-pCLAMP8.1. To record ERGs from a single stimulation, ~1 sec of light flashed using a halogen lamp (~1,700 Lux). To record ERGs from repeated stimulations, repeated cycles of ~1 sec of light followed by ~1.5 sec of darkness was used. See [104] for a detailed method of ERG recording in *Drosophila*.

### Bright Field and TEM

Fly heads were dissected and fixed overnight at 4°C in 4% paraformaldehyde, 2% glutaraldehyde, 0.1 M sodium cacodylate (pH 7.2), postfixed in 1% OsO_4_ for 1 h, dehydrated in ethanol and propylene oxide, and then embedded in Embed-812 resin (Electron Microscopy Sciences). One micron-thick sections were stained with toluidine blue and imaged with a Zeiss microscope (Axio Imager-Z2) equipped with an AxioCam MRm digital camera. Thin sections (~50 nm) were stained in 4% uranyl acetate and 2.5% lead nitrate, and TEM images were captured using a transmission electron microscope (model 1010, JEOL). Images were processed with ImageJ and Adobe Photoshop. See [105] for detailed methods.

### Fluorescence Staining

For immunostaining of larval tissue and adult testis, tissues were dissected in PBS (pH7.2), fixed in 3.7% formaldehyde in PBS for 20 min, and washed in 0.2% Triton X-100 in PBS (PBT). For whole mount staining of fly eyes, heads were prefixed in 4% formaldehyde in PBS for 30 min after removal of the proboscis. Fly eyes were then dissected from these heads, fixed for another 15 min, and washed in 0.3% Triton X-100 in PBS. Fixed samples were blocked in 1X PBS containing 5% normal goat serum and 0.2% Triton X-100 for 1 h (PBTS). Samples were incubated in primary antibody diluted in PBTS overnight at 4^°^C. For anti- PI(4,5)P_2_ staining, samples were incubated in primary antibody for two days at 4^°^C. Samples were washed in PBT, incubated in secondary antibody diluted in PBT for two hours at room temperature, and then washed in PBT prior to mounting. Primary antibodies were used at the following dilutions: Mouse monoclonal anti-Rh1 4C5, DSHB[106] 1:50, Rabbit anti-GFP (Invitrogen) 1:500, mouse anti-ATP synthase α subunit (Complex V; MitoSciences) 1:500, mouse anti- PI(4,5)P_2_ (Echelon) 1:100. Secondary antibodies conjugated to Cy3 (Jackson ImmunoResearch Laboratories, Inc.) or Alexa Fluor 488 (Invitrogen) were used at 1:500. Phalloidin conjugated with Alexa 488 or Alexa 647 (Invitrogen) 1:250 was added with secondary antibody. Samples were mounted in Vectashield (Vector Laboratories) before imaging with a confocal microscope.

### Western Blots

Heads from 1–2-d-old flies were used. Samples were processed as described in [57]. Primary antibodies were used at the following dilutions: rabbit Arr2 (1:2000) [46], rabbit RdgC (1:2000) [107], rabbit Trp (1:2000) [108], rabbit Inad (1:2000) [109], rabbit CalX (1:2000) [110], mouse Rh1(1:2000) DSHB [106], rabbit NinaC (1:1000) [111], rabbit PKC (1:1000) [112], mouse Actin (1:5000) (ICN Biomedicals) and Anti-Bsf (1:1000) [113]. All secondary antibodies conjugated to HRP (Jackson ImmunoResearch Laboratories, Inc.) were used at 1:10,000.

### Quantitative PCR for mtRNA Content

Total RNA was isolated from control and *ppr* ^*A*^ third instar larvae. Five micrograms of total RNA from each sample were reverse transcribed using Random Hexamer Primers and the High-Capacity cDNA Reverse Transcription Kit (Applied Biosystems). RT—qPCR analysis of the rp49, mitochondrial precursor and mature mitochondrial transcripts were performed in triplicates using 150ng of cDNA per reaction on a 7900HT Real-Time PCR System using ABI SYBR Green PCR Master Mix (Applied Biosystems). An initial activation step for 10 min at 95°C was followed by 40 cycles of 95°C for 10 s and 60°C for 30 s. The primer sequences used are provided in S1 Table. Data is presented as mean ± SD. Fold change was calculated as previously described [114], and statistical significance was determined using a two-tailed Student’s *t* test *(p* < 0.05).

### Quantitative PCR for mtDNA Content

Method adopted from [115]. *Drosophila* whole DNA (genomic and mitochondrial) was purified from third instar larvae as the template for PCR. Template DNA was mixed with primers and green supermix reagent (iQ SYBR; Bio-Rad Laboratories). PCR was performed in a thermal cycler (iCycler; Bio-Rad Laboratories), and the data were collected and analyzed using the optical module (iQ5; Bio-Rad Laboratories) and related software following the manufacturer’s instructions. The following primer pairs were used to amplify a genomic DNA fragment corresponding to *CG9277/β-Tubulin* or a mitochondrial DNA fragment corresponding to *CG34083/ND5*, respectively: β-Tubulin forward, 5′-CCTTCCCACGTCTTCACTTC-3′; and β-Tubulin reverse, 5′-TTCTTGGCATCGAACATCTG-3′; and ND5 forward, 5′-GCAGAAACAGGTGTAGGAGCA-3′; and ND5 reverse, 5′-GCTGCTATAACTAAAAGAGCTCAGA-3′. Dissociation curves for the amplicons were generated after each run to confirm that the fluorescent signals were not attributable to nonspecific signals (primer-dimers). The mtDNA content (mtDNA/β-Tubulin ratio) was calculated using the formula: mtDNA content = 1/2^**ΔCt**^, where ΔCt = Ct^**mtDNAΔ**^—Ct^**β-Tubulin**^.

### Mitochondrial Physiology

Enzymatic activity assays were performed on larval whole cell extracts or isolated mitochondria from third instar larvae as previously described [13,116]. Polarography was performed on isolated mitochondria from third instar larvae as previously described [13,117]. Aconitase activity assays were performed in isolated mitochondria from third instar larvae as previously described [13]. ATP level for larvae, eyes, and heads were determined by ATP assay kit (Invitrogen) [118,119]. Flies were exposed to light (~1,800 Lux) for 1 h prior to detection of ATP levels in adult eyes and heads. Eyes were dissected in PBS, and heads were frozen on dry ice and separated on a metal plate kept on dry ice. Five third instar larvae, 20 eyes or 5 heads were dissected and homogenized in 50 μl of 100 mM Tris and 4 mM, EDTA, pH 7.8. These homogenates were snap-frozen in liquid nitrogen and then boiled for 3 min. Samples were then centrifuged, and the supernatant was diluted (1/50 for larvae and 1/2 for heads and eyes) in extraction buffer mixed with luminescent solution. Luminescence was measure on FLUOstar OPTIMA plate reader. DHE staining was performed as described previously [78]. Flies were exposed to 24 h light (1,800 Lux) prior to DHE staining in adult eyes.

### Bioinformatics

Percentage protein similarity was determined using BlastP (NCBI). Protein domains were analyzed by PROSITE [120].

## Supporting Information

S1 Data Excel spreadsheets contain the numerical data. (XLSX)

S1 Fig Mapping of *ppr* alleles. (A) Mapping strategy for *ppr* alleles. First, duplication mapping was performed. The lethality of *ppr* mutant alleles was rescued by duplication D*p(1;Y) y* ^*2*^ *67g19*.*1* (blue, chromosomal location is indicated by dotted lines). Second, complementation tests were performed among mutants rescued by duplication (*Dp(1;Y) y* ^*2*^ *67g19*.*1*). All five *ppr* alleles (shown in Fig 1) failed to complement each other. Molecularly mapped deficiencies were used to narrow down the location of the mutations to a 25 kb region. Third, mutations in *CG14786*/*ppr* were identified by Sanger sequencing of this 25 kb region (shown in Fig 1). Lethality associated with *ppr* alleles was rescued by a 22 kb genomic rescue P[acman] construct (CH322-75O21) as well as a 5 kb genomic rescue transgene, both uncovering the wild-type *CG14786/ppr* sequence. (B) Amino acid sequence of predicted PPR repeat motifs in Ppr protein. (C–D) Colocalization of the GFP-tagged Ppr protein (green) with mitochondrial complex V (ATP5A antibody, red) in adult testes and ommatidia (D). The seven rhabdomeres that can be observed in an ommatidium are stained by Phalloidin/Actin (blue in D).
(TIF)

S2 Fig PR degeneration due to *ppr* loss of function is light dependent. (A–I) Bright field images of retinal sections from control (A, D, G), *ppr* ^*A*^ (B, E, H) and *ppr* ^*E*^ (C, F, I) eye clones. Flies were raised in the dark for one day (A–C), three weeks (D–F), or in a 12 h light/dark cycle for three weeks (G–I).
(TIF)

S3 Fig PI(4,5)P_2_ levels are not altered in *ppr* mutant PRs. (A–C) Anti-PI(4,5)P_2_ immunostaining (green or grey) in rhabdomeres (blue). Rhabdomeres are stained by Phalloidin/Actin. Intense PI(4,5)P_2_ staining is seen following 2 min exposure to blue light in *norpA* ^*P24*^/*PLC* mutant (A), due to its lack of PI(4,5)P_2_-cleaving activity. This is in sharp contrast to the mild PI(4,5)P_2_ staining that is observed in controls under the same conditions (B). No difference in PI(4,5)P_2_ staining was detected between wild-type (-/+, RFP, red) and *ppr* ^*A*^ mutant PRs (lacking RFP,-/-) when exposed to white light for 10 min. (D) Relative ERG amplitude from control, *ppr* ^*A*^, *trp* ^*P365*^/+ and *ppr* ^*A*^; *trp* ^*P365*^/+. *trp* ^*P365*^ mutant encodes a constitutive active Trp channel causing constant Ca^2+^ influx. (E) *trp* ^*P365*^/+ and *ppr* ^*A*^; *trp* ^*P365*^/+ mutant rhabdomeres stained with Phalloidin/Actin (green).
(TIF)

S4 Fig Light induces Rh1 accumulation in *ppr* mutant PRs. (A–D) Rh1 (grey) immunostaining on one micron sections of control (A, C) and *ppr* ^*A*^ mutant (B, D) eyes. The flies used in this experiment were 3–4 d old and raised in the dark (A, B) or exposed to ~30 h of light (C, D). Yellow arrows indicate Rh1 punctae in the cytoplasm (D). (E) Transient blue light exposure converts Rh1 to mRh1, which is phosphorylated and inactivated by Arr2 binding. MRh1, in turn, is recycled to Rh1 by a process that requires (1) an orange photon, (2) Ca^2+^ dependent activation of Retinal Degeneration C (RDGC) to dephosphorylate Rh1- and (3) Ca^2+^-dependent Arr2 release (E, left) [31–34,45]. A reduced Ca^2+^ influx would impair Rh1 dephosphorylation and Arr2 release causing endocytosis of Rh1-Arr2 complex [33,47,51,52]. A 10 min transient exposure to blue light followed by orange light, which is typically required for Rh1 cycling, induces Rh1 (green) internalization in *ppr* mutant (-/-, lacking RFP) but not in control (-/+, marked by RFP) PRs in mosaic eyes. Eyes were fixed and stained upon 24 h of light exposure. Rhabdomeres are stained by Phalloidin/Actin (blue). (F) Colocalization of internalized Rh1 (red) and Arr2::GFP (green), indicated by yellow arrows, in *ppr* mutant PRs (-/-, lack of RFP). Wild-type PRs (-/+, encircled by blue dotted line) are marked by RFP (blue). Flies were exposed to 2 d of light followed by 13 h of darkness. Transient exposure to light during dissection allows translocation of free cytoplasmic Arr2 to rhabdomeres, facilitating visualization of Arr2 that is present in cytoplasmic complexes.
(TIF)

S5 Fig Photoreceptor degeneration in *ppr* is mediated by Rh1 toxicity. (A) Western blot to compare Rh1 levels in eyes from flies raised on normal or low vitamin A food. (B) Bright field images of retinal sections of control and *ppr* ^*A*^ eye clones. Flies were raised in low vitamin A food and kept in the dark for 3 wk (*ppr* ^*A*^, middle) or in a 12 h light/dark cycle for 3 wk (left, control and right, *ppr* ^*A*^). (C) Relative ERG amplitude from control, *ppr* ^*A*^, and *ppr* ^*A*^-expressing Vps35 in R1–R6 using Rh1-Gal4. All flies carried Rh1-GAL4 in this experiment. Flies were raised in constant light for seven days. Error bars represent **±** SEM; statistical significance was determined using a two-tailed Student’s *t* test (*p*-value ***<0.001).
(TIF)

S6 Fig ROS is not increased in *kdn* ^*16A*^ and *pdha* ^*21A*^ mutant cells. (A–B) Mitotic clones of *kdn* ^*16A*^ (A, nongreen cells) or *pdha* ^*21A*^ (B, nongreen cells), marked by loss of GFP in eye imaginal discs. ROS is detected by DHE (red). The yellow dashed lines encircle mutant clones.
(TIF)

S7 Fig Light independent and light dependent PR degeneration due to loss of *sicily*. (A) Relative ATP levels from control and *sicily* ^*7E*^ third instar larval extracts. (B) Quantification of relative ERG amplitude from control and *sicily* ^*7E*^ eye clones. Flies were raised in the dark. Upon eclosion, they were kept in the dark for two (blue) or seven days (red) or constant light for seven days (green). (C) Quantification of relative ERG amplitudes measured during repetitive light stimuli (1 sec light and 1.5 sec dark, as shown in Fig 3A) from control or *sicily* ^*7E*^ eye clones. Error bars represent ± SEM; Student's *t* test (*p*-values: **<0.01, ***<0.001. (D, E) Whole mount Rh1 (red) immunostaining in *sicily* ^*7E*^ mutant PRs. Rhabdomeres are marked by Phalloidin/Actin (green). The flies used in this experiment were 2–3 days old and were either raised in the dark (C) or exposed to ~24 h of light (D) prior to staining.
(TIF)

S1 Table List of primers used for RT-qPCR. (XLSX)

## Abbreviations:

Arr2: Arrestin2
*bsf*: *bicoid stability factor*
CS: citrate synthase
DHE: dihydroethidium
ERG: electroretinograms
ETC: electron transport chain
GFP: Green Fluorescent Protein
hSOD1: human copper-zinc superoxide dismutase
LHON: Leber hereditary optic neuropathy
MRh1: meta-Rh1
OXPHOS: oxidative phosphorylation
P[acman] BAC: (P/ΦC31 artificial chromosome for manipulation)
PI(4,5)P_2_: phosphatidylinositol 4,5-bisphosphate
PLC: Phospholipase C
PPR: pentatricopeptide repeat containing protein
PR: photoreceptor RCR, respiratory control ratio
RDGC: Retinal Degeneration C
Rh1: Rhodopsin1
ROS: reactive oxygen species
SD: standard deviation; SdhA, Succinate dehydrogenase A
SEM: standard error of the mean
TCA: tricarboxylic acid
TEM: Transmission Electron Microscopy
TRP: Transient Receptor Potential
