# Correction: Impaired Mitochondrial Energy Production Causes Light-Induced Photoreceptor Degeneration Independent of Oxidative Stress

**DOI:** 10.1371/journal.pbio.1002622

**Published:** 2018-03-06

**Authors:** Manish Jaiswal, Nele A. Haelterman, Hector Sandoval, Bo Xiong, Taraka Donti, Auinash Kalsotra, Shinya Yamamoto, Thomas A. Cooper, Brett H. Graham, Hugo J. Bellen

A reference is omitted from the first sentence of the second paragraph under the heading “Ppr Localizes to Mitochondria and Its Loss Causes a Progressive Defect in ERGs” in the Results section. The sentence should read:

The causative mutations of the five alleles of this complementation group were mapped to *CG14786/Lrpprc2 (ppr)* (Fig 1B and 1C and S1 Fig), which is required for the coordination of mitochondrial translation (Baggio et al., 2014).

The reference is: Baggio F, Bratic A, Mourier A, Kauppila TE, Tain LS, Kukat C, et al. (2014) *Drosophila melanogaster* LRPPRC2 is involved in coordination of mitochondrial translation. Nucleic Acids Res. Dec 16;42(22):13920–38. doi: 10.1093/nar/gku1132. Epub 2014 Nov 26.

## References

[1] Jaiswal M, Haelterman NA, Sandoval H, Xiong B, Donti T, Kalsotra A, et al (2015) Impaired Mitochondrial Energy Production Causes Light-Induced Photoreceptor Degeneration Independent of Oxidative Stress. PLOS Biology 13(7): e1002197 https://doi.org/10.1371/journal.pbio.1002197 26176594 10.1371/journal.pbio.1002197 PMC4503542

