# Supplementary figures and images for: Impaired Mitochondrial Energy Production Causes Light-Induced Photoreceptor Degeneration Independent of Oxidative Stress
Source: PLoS Biol. 2015 Jul 15;13(7):e1002197. doi: 10.1371/journal.pbio.1002197 (PMC4503542; doi:10.1371/journal.pbio.1002197)

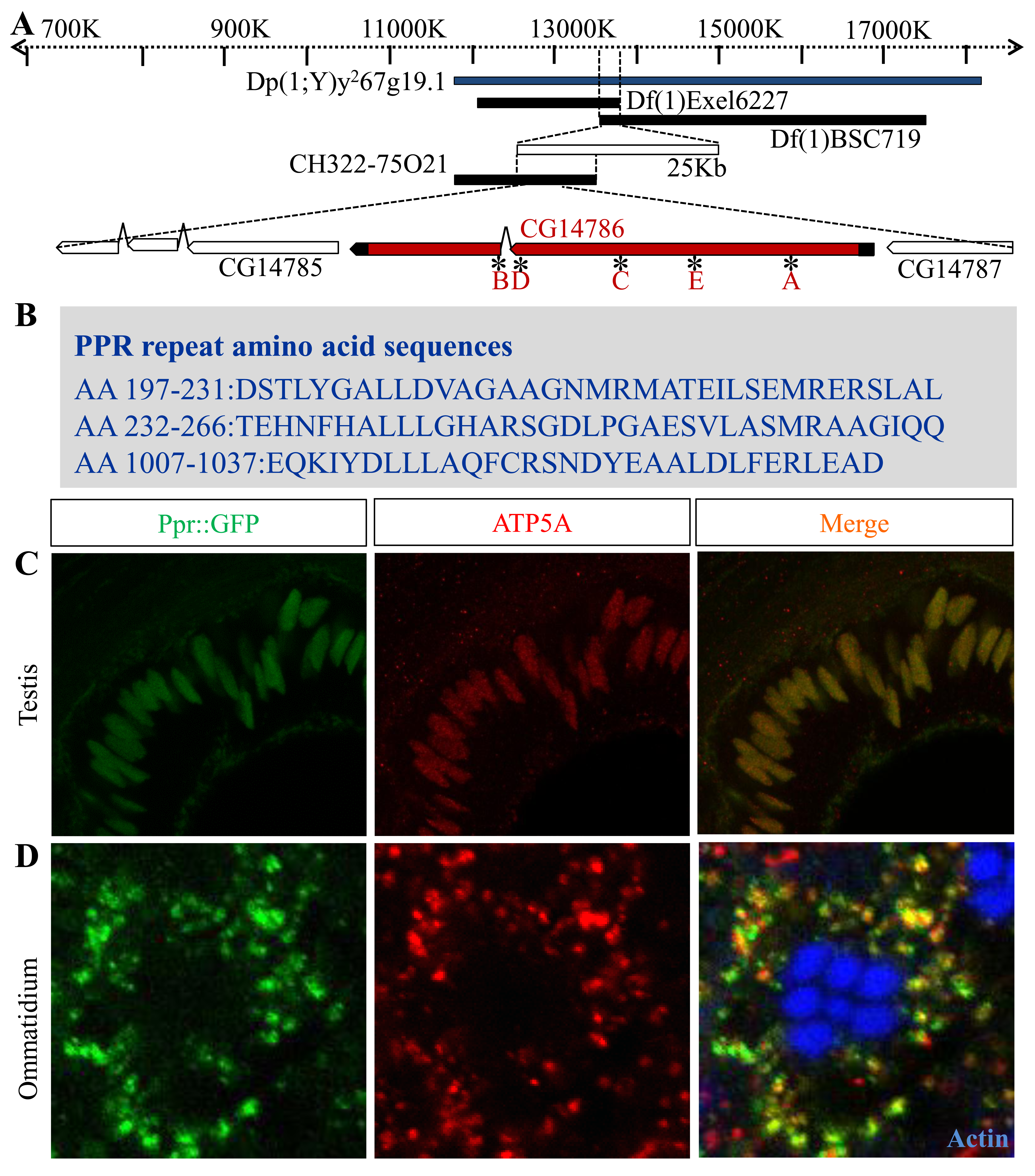

Supplement: S1 Fig — (A) Mapping strategy for ppr alleles. First, duplication mapping was performed. The lethality of ppr mutant alleles was rescued by duplication Dp(1;Y) y 2 67g19.1 (blue, chromosomal location is indicated by dotted lines). Second, complementation tests were performed among mutants rescued by duplication (Dp(1;Y) y 2 67g19.1). All five ppr alleles (shown in Fig 1) failed to complement each other. Molecularly mapped deficiencies were used to narrow down the location of the mutations to a 25 kb region. Third, mutations in CG14786/ppr were identified by Sanger sequencing of this 25 kb region (shown in Fig 1). Lethality associated with ppr alleles was rescued by a 22 kb genomic rescue P[acman] construct (CH322-75O21) as well as a 5 kb genomic rescue transgene, both uncovering the wild-type CG14786/ppr sequence. (B) Amino acid sequence of predicted PPR repeat motifs in Ppr protein. (C–D) Colocalization of the GFP-tagged Ppr protein (green) with mitochondrial complex V (ATP5A antibody, red) in adult testes and ommatidia (D). The seven rhabdomeres that can be observed in an ommatidium are stained by Phalloidin/Actin (blue in D). (TIF) [file pbio.1002197.s002.tif]

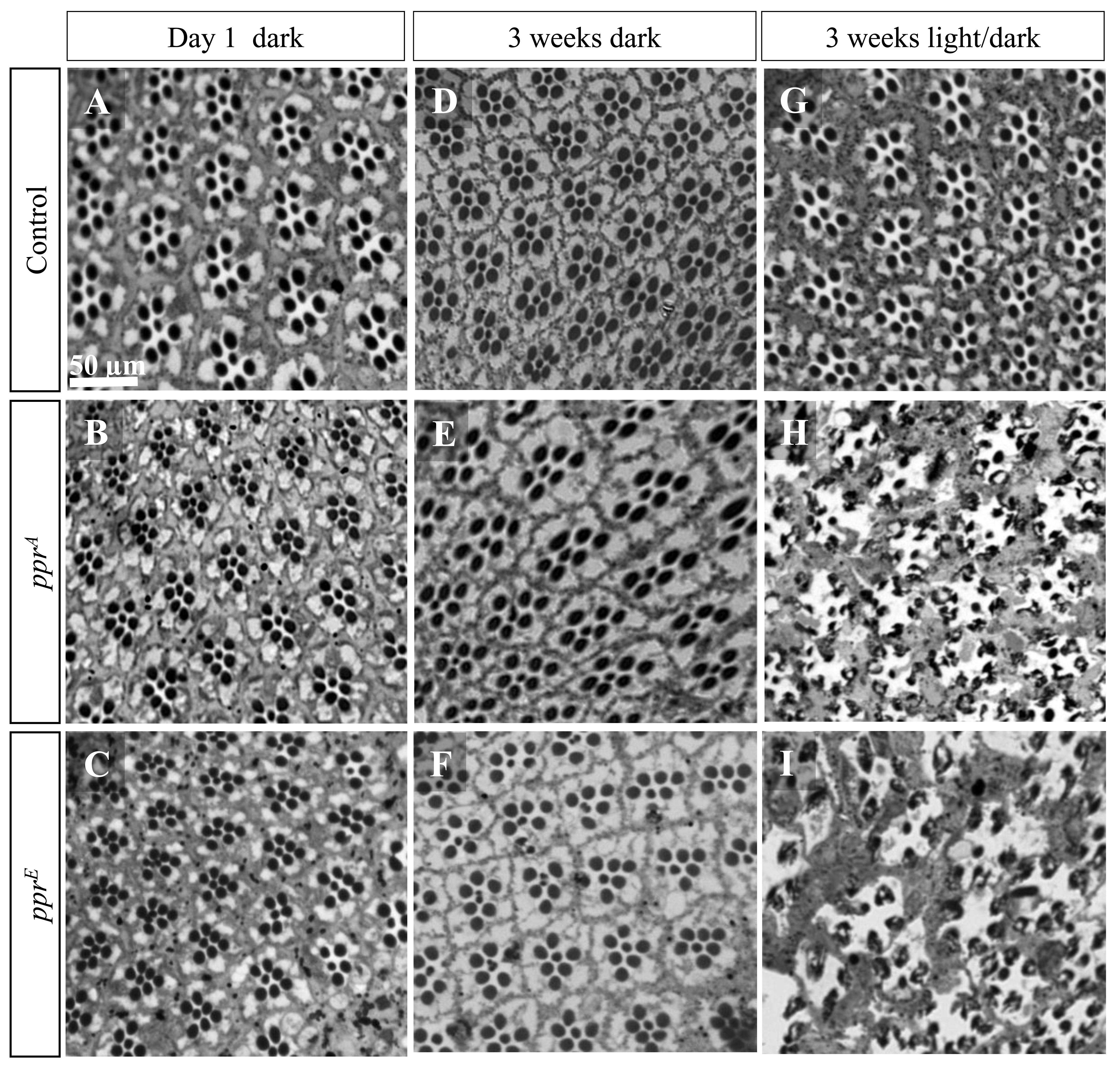

Supplement: S2 Fig — (A–I) Bright field images of retinal sections from control (A, D, G), ppr A (B, E, H) and ppr E (C, F, I) eye clones. Flies were raised in the dark for one day (A–C), three weeks (D–F), or in a 12 h light/dark cycle for three weeks (G–I). (TIF) [file pbio.1002197.s003.tif]

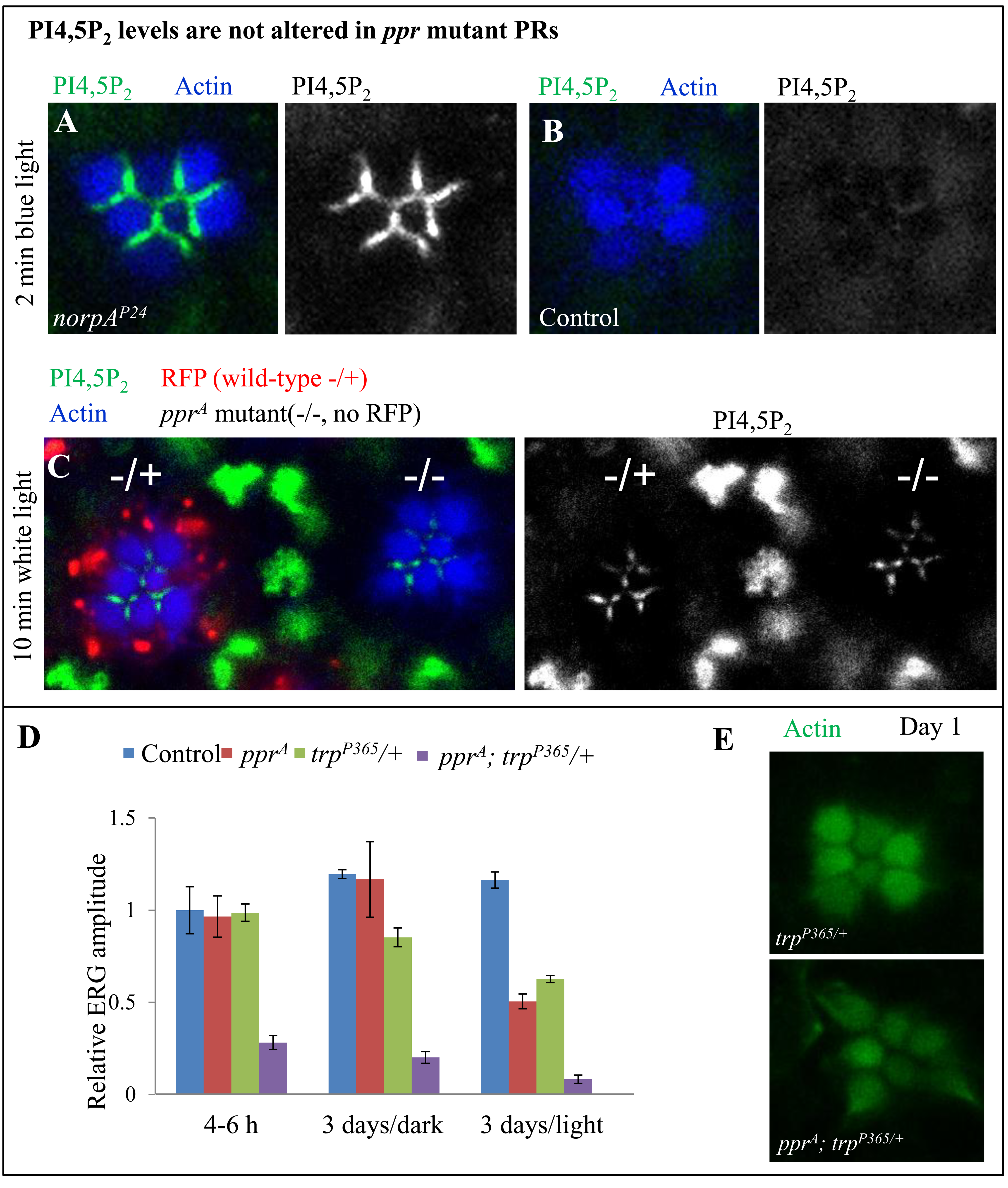

Supplement: S3 Fig — (A–C) Anti-PI(4,5)P2 immunostaining (green or grey) in rhabdomeres (blue). Rhabdomeres are stained by Phalloidin/Actin. Intense PI(4,5)P2 staining is seen following 2 min exposure to blue light in norpA P24/PLC mutant (A), due to its lack of PI(4,5)P2-cleaving activity. This is in sharp contrast to the mild PI(4,5)P2 staining that is observed in controls under the same conditions (B). No difference in PI(4,5)P2 staining was detected between wild-type (-/+, RFP, red) and ppr A mutant PRs (lacking RFP,-/-) when exposed to white light for 10 min. (D) Relative ERG amplitude from control, ppr A, trp P365/+ and ppr A; trp P365/+. trp P365 mutant encodes a constitutive active Trp channel causing constant Ca2+ influx. (E) trp P365/+ and ppr A; trp P365/+ mutant rhabdomeres stained with Phalloidin/Actin (green). (TIF) [file pbio.1002197.s004.tif]

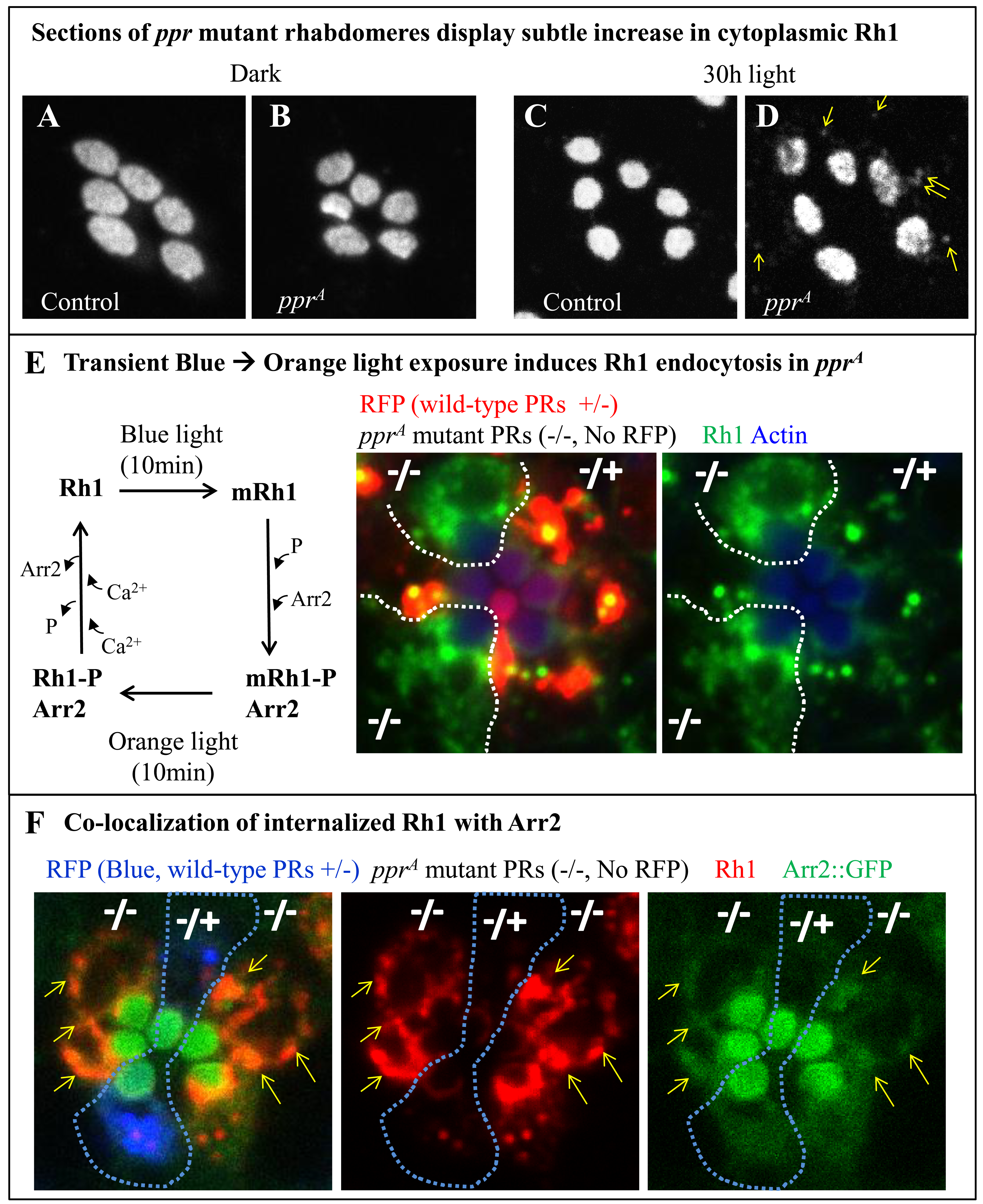

Supplement: S4 Fig — (A–D) Rh1 (grey) immunostaining on one micron sections of control (A, C) and ppr A mutant (B, D) eyes. The flies used in this experiment were 3–4 d old and raised in the dark (A, B) or exposed to ~30 h of light (C, D). Yellow arrows indicate Rh1 punctae in the cytoplasm (D). (E) Transient blue light exposure converts Rh1 to mRh1, which is phosphorylated and inactivated by Arr2 binding. MRh1, in turn, is recycled to Rh1 by a process that requires (1) an orange photon, (2) Ca2+ dependent activation of Retinal Degeneration C (RDGC) to dephosphorylate Rh1- and (3) Ca2+-dependent Arr2 release (E, left) [31–34,45]. A reduced Ca2+ influx would impair Rh1 dephosphorylation and Arr2 release causing endocytosis of Rh1-Arr2 complex [33,47,51,52]. A 10 min transient exposure to blue light followed by orange light, which is typically required for Rh1 cycling, induces Rh1 (green) internalization in ppr mutant (-/-, lacking RFP) but not in control (-/+, marked by RFP) PRs in mosaic eyes. Eyes were fixed and stained upon 24 h of light exposure. Rhabdomeres are stained by Phalloidin/Actin (blue). (F) Colocalization of internalized Rh1 (red) and Arr2::GFP (green), indicated by yellow arrows, in ppr mutant PRs (-/-, lack of RFP). Wild-type PRs (-/+, encircled by blue dotted line) are marked by RFP (blue). Flies were exposed to 2 d of light followed by 13 h of darkness. Transient exposure to light during dissection allows translocation of free cytoplasmic Arr2 to rhabdomeres, facilitating visualization of Arr2 that is present in cytoplasmic complexes. (TIF) [file pbio.1002197.s005.tif]

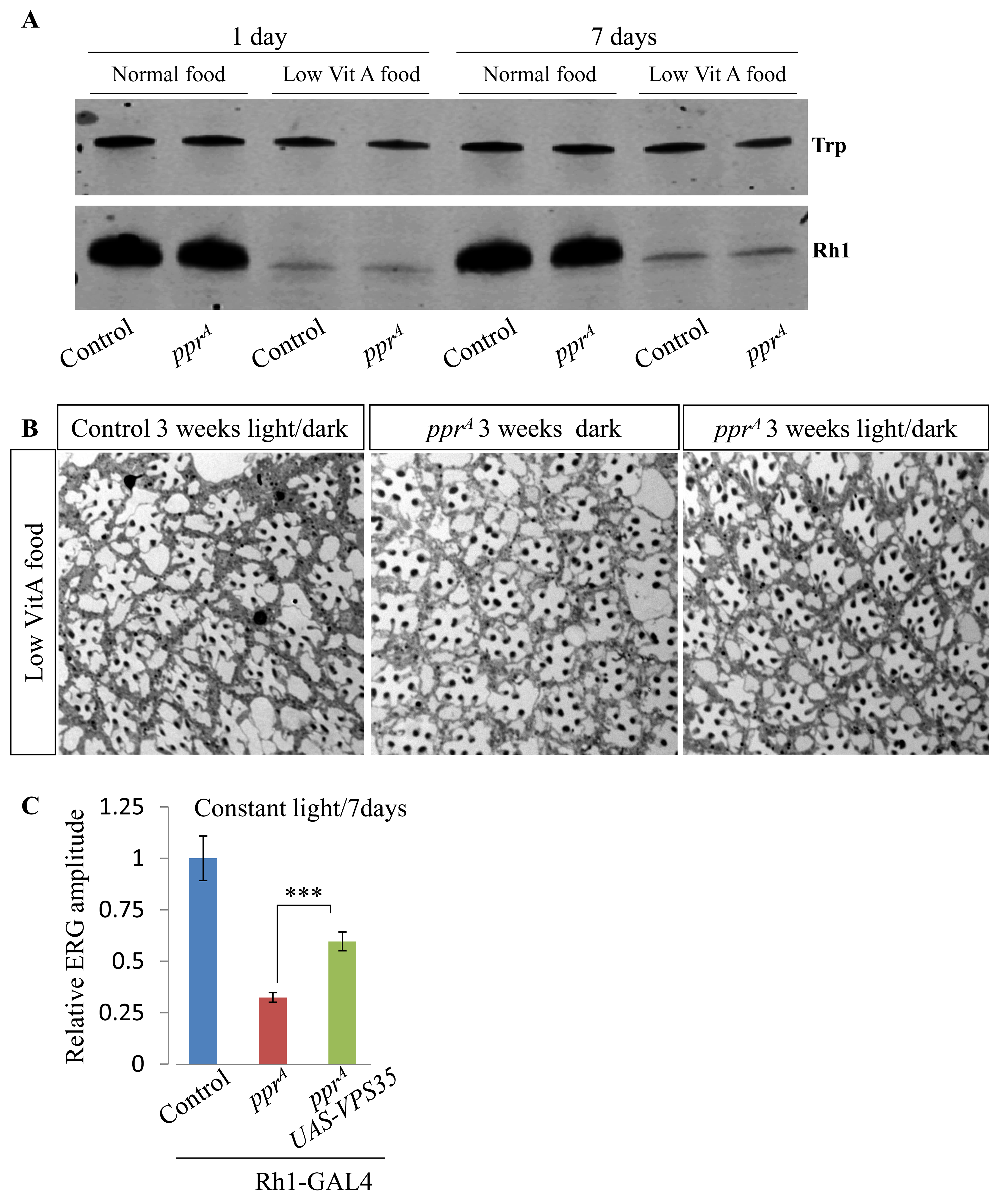

Supplement: S5 Fig — (A) Western blot to compare Rh1 levels in eyes from flies raised on normal or low vitamin A food. (B) Bright field images of retinal sections of control and ppr A eye clones. Flies were raised in low vitamin A food and kept in the dark for 3 wk (ppr A, middle) or in a 12 h light/dark cycle for 3 wk (left, control and right, ppr A). (C) Relative ERG amplitude from control, ppr A, and ppr A-expressing Vps35 in R1–R6 using Rh1-Gal4. All flies carried Rh1-GAL4 in this experiment. Flies were raised in constant light for seven days. Error bars represent ± SEM; statistical significance was determined using a two-tailed Student’s t test (p-value ***<0.001). (TIF) [file pbio.1002197.s006.tif]

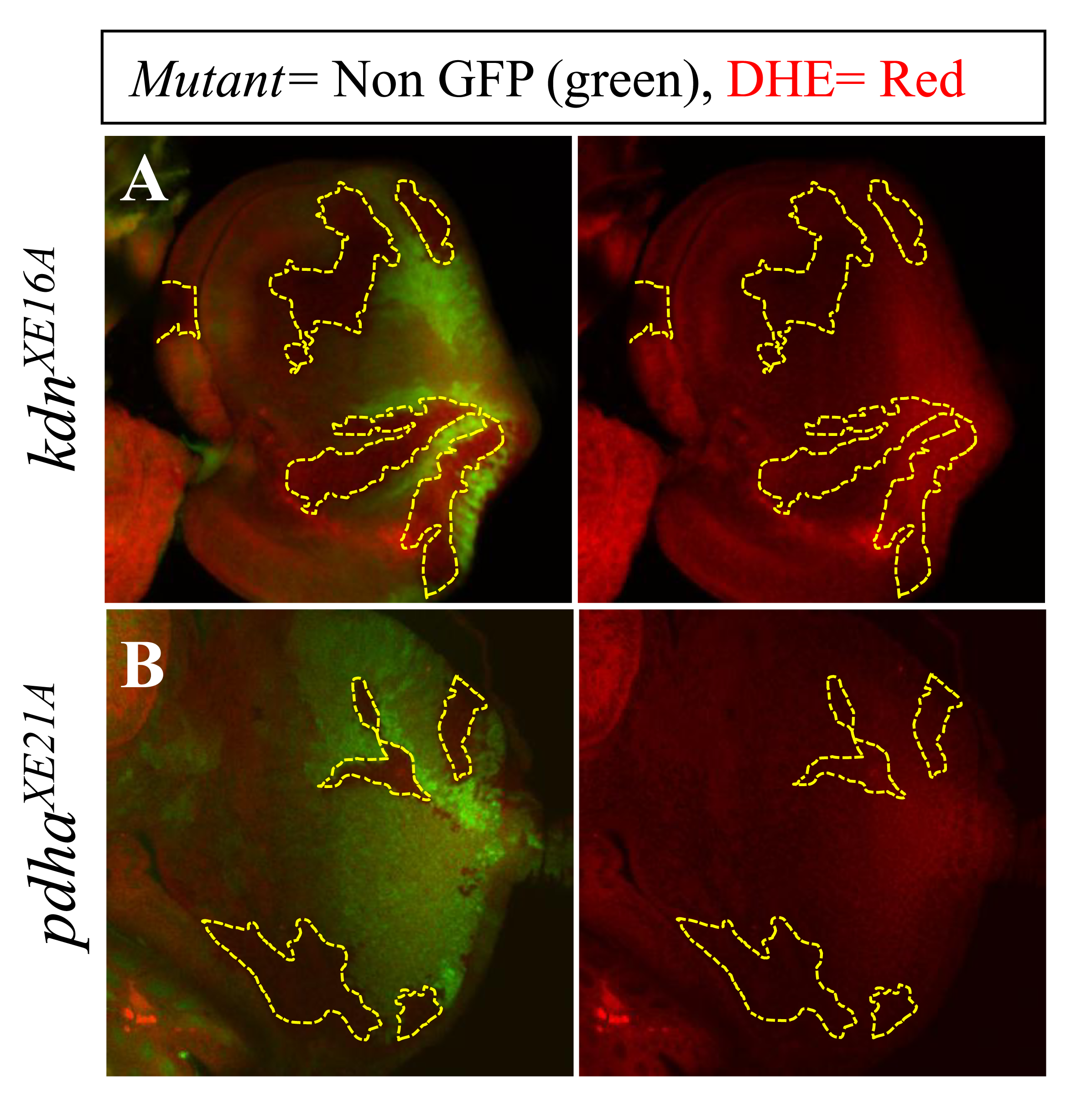

Supplement: S6 Fig — (A–B) Mitotic clones of kdn 16A (A, nongreen cells) or pdha 21A (B, nongreen cells), marked by loss of GFP in eye imaginal discs. ROS is detected by DHE (red). The yellow dashed lines encircle mutant clones. (TIF) [file pbio.1002197.s007.tif]

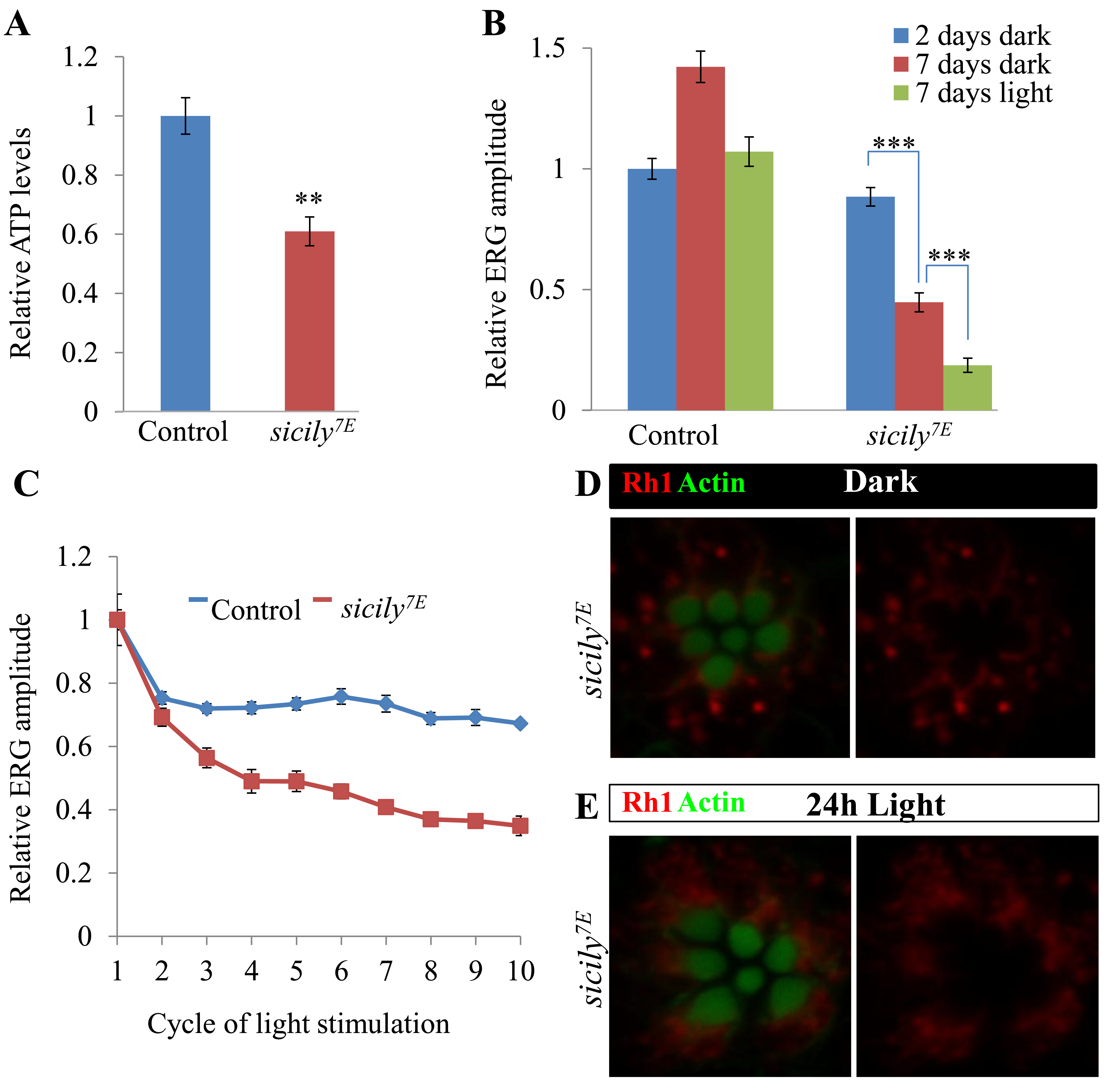

Supplement: S7 Fig — (A) Relative ATP levels from control and sicily 7E third instar larval extracts. (B) Quantification of relative ERG amplitude from control and sicily 7E eye clones. Flies were raised in the dark. Upon eclosion, they were kept in the dark for two (blue) or seven days (red) or constant light for seven days (green). (C) Quantification of relative ERG amplitudes measured during repetitive light stimuli (1 sec light and 1.5 sec dark, as shown in Fig 3A) from control or sicily 7E eye clones. Error bars represent ± SEM; Student's t test (p-values: **<0.01, ***<0.001. (D, E) Whole mount Rh1 (red) immunostaining in sicily 7E mutant PRs. Rhabdomeres are marked by Phalloidin/Actin (green). The flies used in this experiment were 2–3 days old and were either raised in the dark (C) or exposed to ~24 h of light (D) prior to staining. (TIF) [file pbio.1002197.s008.tif]
